# Staged miRNA re-regulation patterns during reprogramming

**DOI:** 10.1186/gb-2013-14-12-r149

**Published:** 2013-12-31

**Authors:** Christine M Henzler, Zhonghan Li, Jason Dang, Mary Luz Arcila, Hongjun Zhou, Jingya Liu, Kung-Yen Chang, Danielle S Bassett, Tariq M Rana, Kenneth S Kosik

**Affiliations:** 1 Neuroscience Research Institute and Department of Cellular, Molecular and Developmental Biology, University of California, Santa Barbara, CA 93106, USA; 2 Program for RNA Biology, Sanford-Burnham Medical Research Institute, La Jolla, CA 92037, USA; 3 Department of Physics, University of California, Santa Barbara, CA 93106, USA; 4 Sage Center for the Study of the Mind, University of California, Santa Barbara, CA 93106, USA; 5 Current address: Minnesota Supercomputing Institute, University of Minnesota, Minneapolis, MN 55455, USA; 6 Current address: Department of Bioengineering, University of Pennsylvania, Philadelphia, PA 19104, USA

## Abstract

**Background:**

MiRNAs often operate in feedback loops with transcription factors and represent a key mechanism for fine-tuning gene expression. In transcription factor-induced reprogramming, miRNAs play a critical role; however, detailed analyses of miRNA expression changes during reprogramming at the level of deep sequencing have not been previously reported.

**Results:**

We use four factor reprogramming to induce pluripotent stem cells from mouse fibroblasts and isolate FACS-sorted Thy1- and SSEA1+ intermediates and Oct4-GFP+ induced pluripotent stem cells (iPSCs). Small RNAs from these cells, and two partial-iPSC lines, another iPSC line, and mouse embryonic stem cells (mES cells) were deep sequenced. A comprehensive resetting of the miRNA profile occurs during reprogramming; however, analysis of miRNA co-expression patterns yields only a few patterns of change. Dlk1-Dio3 region miRNAs dominate the large pool of miRNAs experiencing small but significant fold changes early in reprogramming. Overexpression of Dlk1-Dio3 miRNAs early in reprogramming reduces reprogramming efficiency, suggesting the observed downregulation of these miRNAs may contribute to reprogramming. As reprogramming progresses, fewer miRNAs show changes in expression, but those changes are generally of greater magnitude.

**Conclusions:**

The broad resetting of the miRNA profile during reprogramming that we observe is due to small changes in gene expression in many miRNAs early in the process, and large changes in only a few miRNAs late in reprogramming. This corresponds with a previously observed transition from a stochastic to a more deterministic signal.

## Background

Deep sequencing technologies have opened numerous windows into the mechanisms driving cell biological phenomena. Sequencing at the RNA level quantifies the transcriptome in a non-biased manner and, when applied to a temporal series of cellular transitions, can detect co-expression networks that suggest functional modules. Reprogramming of mouse embryonic fibroblasts (MEFs) to induced pluripotent stem cells (iPSCs) is one such series of cellular transitions and is of enormous interest to biologists. Reprogramming can be staged as an epigenetic series of gene expression changes that begins with downregulation of fibroblast genes followed by induction of temporally controlled mouse embryonic stem (mES) cell markers, activation of endogenous mES self-renewal genes, and the establishment of mES gene regulation networks [1,2]. Reprogramming epochs can be marked by the loss of the membrane glycoprotein Thy1 immunoreactivity as fibroblasts shed their identity, followed by activation of the pluripotency markers alkaline phosphatase (AP) and SSEA1 [1,2], and then activation of embryonic stem cell factor genes such as *Oct4*, *Sox2*, *Klf4*, *Nanog* and *Sall4*[3-5]. Failure to suppress differentiation-associated genes or block differentiation signals leads to incomplete reprogramming [6].

More detailed analyses have shown that the immediate response to the reprogramming factors includes upregulation of mesenchymal-to-epithelial transition (MET) genes [7,8] and proliferation genes, consistent with both *c-Myc* expression [6] and the requirement to overcome the barrier of cell-cycle arrest early [9,10]. These and other studies highlight the patterns of mRNA expression induced by single or multiple reprogramming factors; however, microRNA (miRNA) expression patterns have received less attention. Polo *et al.*[11] used microarrays to investigate miRNA expression patterns in later stages of reprogramming, but the role of miRNAs early in reprogramming remains incompletely defined.

miRNAs are highly accurate markers of cell identity (reviewed in [12]). Their profiles unambiguously distinguish cell types, including embryonic stem cells [13,14], a vast variety of precursor cells, terminally differentiated cells, and tumor types, even among closely related cancers [15]. miRNAs play important functional roles in stem cells, including the regulation of pluripotency, self-renewal and reprogramming of somatic cells (reviewed in [16]).

To investigate the overall pattern of miRNA expression during reprogramming, we deeply sequenced the small RNA population of mouse embryonic fibroblasts during reprogramming. These datasets were analyzed by two complementary statistical techniques - one to identify differentially expressed miRNAs and the other to detect putatively co-regulated modules. The analysis identified unique miRNA expression signatures among reprogramming intermediates as well as the cell lines that failed to achieve pluripotency. Deep sequencing’s large dynamic range and ability to detect all expressed miRNAs demonstrated with high resolution that large numbers of miRNAs undergo significant changes in expression during reprogramming. We have identified sets of miRNAs that undergo expression changes at transition points and show that these miRNAs appear to be expressed as modules with unique expression patterns, some with annotated functional assignments. A staged expression pattern was observed in which small fold changes among a large number of miRNAs occur at the earliest time point in reprogramming and this pattern shifts to large fold changes among a small number of miRNAs as reprogramming progresses. A recent report analyzed reprogramming at the single-cell level and found that gene expression between sister cells varied greatly [17]. They proposed that stochasticity was characteristic of the early stage of reprogramming. Following this phase, cells destined for pluripotency demonstrated a more uniform and predictable sequence of gene expression changes referred to as a 'hierarchical mechanism' [17]. The pattern of lower magnitude changes in miRNA levels observed during the very early phase of reprogramming followed by later stages characterized by large changes in only a few miRNAs coincides with a stochastic early phase followed by a later deterministic phase.

## Results

### miRNAs identify distinct reprogramming stages

**Figure 1:**
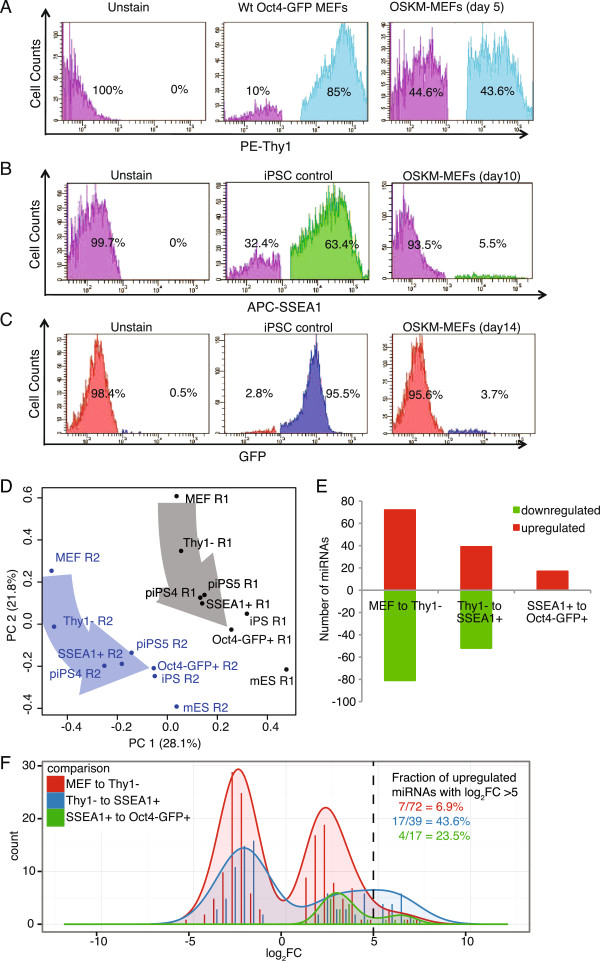
**Sample isolation and overall miRNA expression patterns. (A)** Collection of the Thy1- population from day 5 cultures of OSKM-infected MEFs. MEFs were transduced with OSKM virus supernatants for 5 days, harvested, and stained with phycoerythrin (PE)-conjugated anti-mouse Thy1 antibody. Wt, wild type **(B)** Collection of the SSEA1+ population from day 10 cultures of OSKM-infected MEFs. OSKM-transduced MEFs were stained with APC-conjugated anti-mouse SSEA1 antibody. **(C)** Collection of the GFP + population from day 14 cultures of OSKM-infected MEFs. Cells were sorted based on endogenous expression of GFP driven by the *Oct4* promoter. **(D)** Principal component (PC) analysis of the 290 most variant miRNAs. Replicate 1 is in black, replicate 2 is in blue. Arrows show the trajectory of reprogramming in each replicate. **(E)** miRNAs differentially expressed (false discovery rate of 5%) during reprogramming in stage to stage transitions. **(F)** Histogram and overlaid density plot of distribution of log_2_ fold changes (FC) calculated by edgeR for the differentially expressed miRNAs in E). The fraction of upregulated miRNAs with a log_2_ fold change >5 in each transition is shown. See also Figures S1 and S2 in Additional file 1.

Intermediates of four factor reprogramming (Oct4, Sox2, Klf4, c-Myc, abbreviated OSKM) [2] in mouse embryonic fibroblasts were used to prepare the small non-coding RNA fraction for deep sequencing and mapping to miRNA sequences (Figure 1A-C; Figure S1 in Additional file 1). The intermediary transitions analyzed were the loss of the fibroblast marker Thy1 (44.6% of fluorescence activated cell sorting (FACS)-sorted cells at day 5) followed by the appearance of the SSEA1 marker (5.5% of cells at day 10), followed, in turn, by expression of endogenous *Oct4* (also known as *Pou5f1*; 3.7% of cells at day 14). Eight cell populations were chosen for deep sequencing: (1) Oct4-GFP MEFs; (2) FACS-sorted Thy1- cells from cultures at day 5 after OSKM (4F) transduced MEFs; (3) sorted SSEA1+ cells harvested at day 9 to 10 post-OSKM; (4) Oct4-GFP+ cells at day 14 post-OSKM; (5) an established iPSC line (iPSC93-2 [18]) generated in the same way as the cells reprogrammed here; (6) an mES cell line, CCE [19]; and (7 and 8) two established partial iPSC lines (cells trapped in an intermediate state). These two partial iPSC lines have morphologies similar to embryonic stem cells, but have not activated endogenous self-renewal markers and remain Oct4-GFP-negative (Figure S1 in Additional file 1).

Reads from these samples mapped to 892 individual miRNA arms. miRBase version 16 contains 580 well-authenticated mouse miRNA hairpins; however, many hairpins have significant numbers of reads from both strands [20]. We limited the dataset to the 581 individual miRNA arms with at least 4 counts per million (cpm) in at least one library in each of the two replicates. Of the 581 miRNAs used to assess differential expression across all samples (Additional file 2), 207 were differentially expressed between MEFs and stem cells (Oct4-GFP+, iPSCs, and mES cells; false discovery rate (FDR) of 5%). This large fraction of miRNAs that undergo changes in expression indicates that a major restructuring of miRNA expression patterns occurs during reprogramming.

To detect collective variation among the samples, the top 290 most variant miRNAs from all 16 samples were analyzed by principal component analysis (PCA; Figure 1D; Figure S2 in Additional file 1), which can capture miRNA expression in low-dimensional space. As observed in Polo *et al.*[11], this non-biased analysis revealed clusters that corresponded to each of the cell types. Thus, miRNAs have the property of defining cell identity among reprogramming intermediates. Furthermore, the profiles from the reprogramming intermediates (MEF, Thy1-, SSEA1+, Oct4-GFP+) were spread along a trajectory that corresponded to the progression of reprogramming.

### A miRNA profile can distinguish the state of partially reprogrammed lines

Partially reprogrammed iPSCs (piPSCs) are stable cell lines trapped at intermediate stages of reprogramming that have silenced the somatic program but failed to activate the pluripotency program [6,21]. By PCA, piPSCs clustered most closely to the SSEA1+ intermediates (Figure 1D). To identify miRNAs that were differentially expressed between the piPSC lines compared to fully reprogrammed iPSC lines and embryonic stem cells, the entire data set was statistically analyzed by edgeR [22]. edgeR identified 87 miRNAs that were differentially expressed between the piPSC lines compared to fully reprogrammed iPSC lines and embryonic stem cells. The pluripotency-associated 106a ~ 363 and 290 ~ 295 clusters were among the most significantly differentially expressed miRNAs between these two clusters, and were all elevated in stem cells compared to piPSCs, indicating that basic changes necessary for establishing pluripotency have not been established in the piPSC lines. miRNAs associated with the MET (miR-200a, b, c-3p, miR-141-3p, miR-429-3p, miR-205-5p) [23-26] were not differentially expressed between the piPSC lines and the stem cell lines, with the exception of miR-200c-3p. In agreement with their placement in the PCA, these piPSC lines appear stuck in the vicinity of the SSEA1+ stage, as the MET miRNAs are significantly upregulated between Thy1- and SSEA1+ stages (see below), and the pluripotency-associated 106a ~ 363 and 290 ~ 295 clusters do not exhibit a pronounced increase in expression until the transition from SSEA1+ to Oct4-GFP+.

### miRNA expression changes at key reprogramming transition points

To assess miRNA changes at successive stages of reprogramming, the re-programmed series alone (MEFs versus Thy1-, Thy1- versus SSEA1+, or SSEA1+ versus Oct4-GFP+) was analyzed by edgeR; 543 miRNAs had at least 4 cpm in at least one of the reprogramming libraries in each replicate. Many (325 at an FDR of 5%) of the 543 miRNAs included in the edgeR analysis were differentially expressed either over the entire MEF to Oct4-GFP+ time series (MEF versus Thy1-, MEF versus SSEA1+, MEF versus Oct4-GFP+), or in the individual stage-to-stage transitions (Figure 1E). This observation again points to the massive resetting of miRNA levels associated with reprogramming. The largest number of differentially expressed miRNAs occurred during the MEF to Thy1- transition, with 81 miRNAs downregulated and 72 upregulated (Additional file 3). In the Thy1- to SSEA1+ transition, 52 miRNAs were downregulated, and 39 were upregulated. From SSEA1+ to Oct4-GFP+ cells, no miRNAs were downregulated and 17 were upregulated.

While the MEF to Thy1- transition had the highest number of differentially expressed miRNAs, the fold changes, albeit significant, were all relatively modest whereas the Thy1- to SSEA1+ and SSEA1+ to Oct4-GFP+ transitions had a larger fraction of miRNAs with large, positive log_2_ fold changes (Figure 1F). This pattern of multiple miRNAs with small fold changes early in reprogramming followed by a much lower number of miRNAs with large fold changes later in reprogramming could contribute to the transition from stochastic to deterministic behavior [17].

### *Dlk1*-*Dio3* miRNAs dominate the earliest changes and undergo isomiR switching

**Figure 2:**
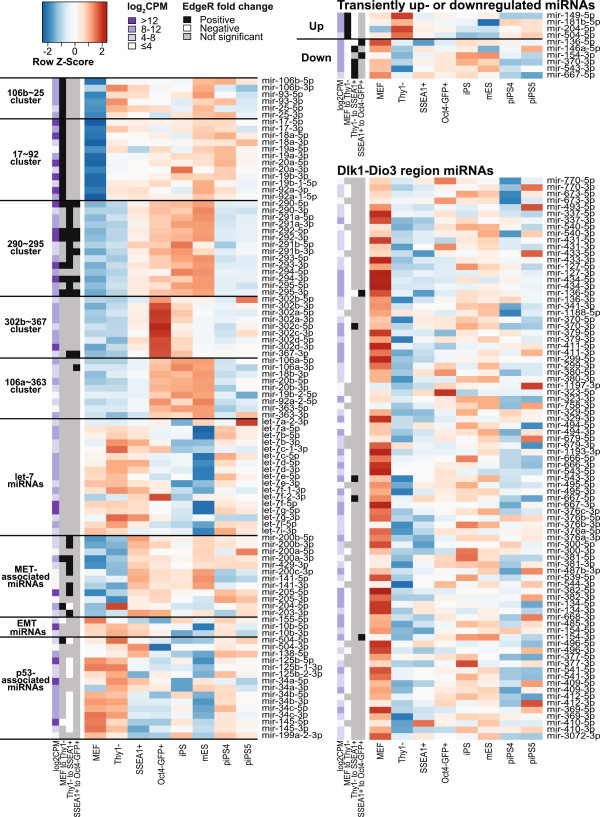
**Heatmap of relative expression of key miRNAs.** Log_2_(normalized counts per million) are centered and scaled by row. For clarity, expression data from only the second replicate are shown. Sidebars show average log_2_ cpm across all 16 treatments and significant fold changes at each transition between stages of reprogramming as determined by edgeR. *Dlk1*-*Dio3* miRNAs are shown in order from 5′ to 3′. EMT, epithelial-mesenchymal transition. See also Figure S3 in Additional file 1.

*Dlk1*-*Dio3* is an imprinted region activated in fully pluripotent mouse stem cells [27,28]. This region contains one of the largest miRNA clusters in the genome [27,29], comprising 59 miRNA hairpins in miRBase v.16. *Dlk1*-*Dio3* protein-coding genes are expressed from the paternal allele, while miRNAs and other non-coding RNAs are expressed from the maternal allele. The miRNAs may be processed from only one or a few transcripts [30]. During the earliest transition in reprogramming, the transition from MEF to Thy1-, most of the downregulated miRNAs lie within the *Dlk1*-*Dio3* gene region. Of the 81 miRNAs significantly downregulated at an FDR of 5%, 66 were in the *Dlk1*-*Dio3* cluster, out of a total of 85 miRNAs in that region in the dataset. One of the 66 was miR-134, which can target *Nanog* and *LRH1*, two transcription factors that upregulate *Oct4*[31]. The 19 *Dlk1*-*Dio3* miRNAs that were not differentially expressed during this transition were generally poorly expressed. As reprogramming proceeds, five of these miRNAs from the *Dlk1*-*Dio3* region were then upregulated at the Thy1- to SSEA1+ and/or SSEA1+ to Oct4-GFP+ transitions (Figure 2). By the Oct4-GFP+ stage, only 35 of the *Dlk1*-*Dio3* miRNAs were still significantly downregulated when compared to starting MEFs. Transient expression changes such as those that occurred with the five *Dlk1*-*Dio3* miRNAs were not common during reprogramming. Only 10 miRNAs underwent significant up- or downregulation during one of the early stage-to-stage transitions of reprogramming, followed by a significant change in expression in the opposite direction in one of the later stages of reprogramming (Figure 2). Six miRNAs were temporarily downregulated during reprogramming, five of which are encoded in the *Dlk1*-*Dio3* locus. All six were significantly downregulated at the MEF to Thy1- transition and then significantly upregulated during either the Thy1- to SSEA1+ (four miRNAs) or SSEA1+ to Oct4-GFP+ (two miRNAs) transitions.

IsomiR switching has the potential to change the mRNA targets of a miRNA especially when the switch occurs within the seed region. miR-485-3p, which is located in the *Dlk1*-*Dio3* region, was the only miRNA with a greater than one nucleotide shift in the 5′ start site in more than 10% of reads. In iPSCs and mES cells, 40 to 70% of the miR-485-3p reads match the canonical sequence, whereas in MEFs 60% of the reads are shifted three bases at the 5′ start site (Figure S3A in Additional file 1). Although many isomiRs work cooperatively with the dominant mature miRNA by repressing similar targets [32], the dominant isomiR in the MEF samples radically changed the predicted targets. TargetScan analysis yielded only four predicted targets; CCR4-NOT transcription complex, subunit 2 (*Cnot2*), G protein-coupled receptor 85 (*Gpr85*), hexamethylene bis-acetamide inducible 1 (*Hexim1*) and IKAROS family zinc finger 2 (*Ikzf2*) overlap between the canonical start site targets and the isomiR in MEFs.

### Expression of MET miRNAs predominates at the transition to SSEA1+ colonies

The MET occurs as colonies become SSEA1+ [7,8]. Concomitant with the large induction of epithelial-associated genes and repression of mesenchymal regulators, MET-associated miRNAs miR-205-5p and the miR-200 family (miR-200a-3p, miR-200b-3p, miR-200c-3p, miR-141-3p and miR-429-3p) [23-26] were markedly upregulated (at least four-fold) in the Thy1- to SSEA1+ transition (Figure 2; Additional file 3). The miR-200 family did not significantly decline in the newly reprogrammed Oct4-GFP+ line; however, many of these miRNAs were expressed at lower levels in the iPSC line with higher passages and in the mES cell line, suggesting that, with additional passages, iPSCs more closely resemble mES cells. miR-181b-5p and miR-204-5p are involved in the transforming growth factor (TGF)β pathway [33,34], and their transient spike at the Thy1- stage corresponds to MET entry. Another TGFβ pathway miRNA, miR-203-3p, was not upregulated until the Thy1- to SSEA1+ transition, along with the miR-200 family [35].

### Expression pattern of stem cell miRNAs during reprogramming

The miRNAs from the mouse stem cell specific 290 ~ 295 cluster have been reported to represent up to 70% of total miRNA reads in deep-sequencing of embryonic stem cells [36]. The 290 ~ 295 cluster begins to increase at the MEF to Thy1- transition and continues to increase throughout reprogramming, reaching its highest levels at the Oct4-GFP+ stage (Figure 2). As shown in previous work [18], the 290 ~ 295 paralogous clusters (clusters 17 ~ 92, 106b ~ 25, 106a ~ 363 and 302b ~ 367) were all highly upregulated in reprogramming (Figure 2; Additional file 3); however, the miRNA 17 ~ 92 and 106b ~ 25 clusters were among the most significantly upregulated miRNAs in the first (MEF to Thy1-) transition, while the 106a ~ 363 and 302b ~ 367 clusters were not significantly upregulated at any single transition (except miR-367-3p at the Thy1- to SSEA1+ transition), but were highly significantly upregulated overall, and in general exhibited a pattern similar to the 290 ~ 295 cluster.

The let-7 family counters the effect of the 290 ~ 295 cluster [37] by inhibiting self-renewal genes. Three of the eight mature let-7 miRNAs, let-7b-5p, let-7e-5p and let-7i-5p, showed significant downregulation across the entire series from MEF to Oct4-GFP+. No let-7 miRNAs showed a significant decrease in any of the stage-to-stage transitions (Figure 2; Additional file 3).

The p53 tumor suppressor pathway is deeply involved in stem cell differentiation, the inhibition of reprogramming and embryonic stem cell self-renewal (reviewed in [38]). p53’s effect on stem cell reprogramming is mediated through multiple miRNAs, as well as p21. Of the p53-upregulated miRNAs, miR-34a/b/c-5p [39], miR-145-5p [40], miR-199a-2-3p [41], miR-34b-5p, miR-34c-5p and miR-145-5p, but not miR-34a-5p and miR-199a-2-3p, showed at least two-fold reduced expression at the Thy1- to SSEA1+ transition.

### Detection of communities of co-expressed miRNAs

Many of the differentially expressed miRNAs have no known functional role in reprogramming. To determine whether miRNAs of unknown function might be co-regulated with miRNAs that have established roles in reprogramming we selected a subset of miRNAs known from the literature to be involved in reprogramming (known reprogramming dataset, KR; Additional file 4). This subset consisted of 125 miRNAs differentially expressed between MEFs and iPSCs or mES cells in mice or humans (KR dataset; Additional file 4). Of these 125 miRNAs, 120 met the criteria for edgeR analysis, and of these, 102 (FDR of 5%) were differentially expressed in our data. Using community detection techniques [42,43], we built a co-regulatory network that divided these 120 miRNAs into co-expression modules (Supplementary Methods in Additional file 1). We then carried out the same network analysis with the larger number of miRNAs with unknown functions that were differentially expressed during reprogramming (at FDRs of 5% and a more stringent 1%) to assess whether they clustered in the same modules as the more thoroughly annotated miRNAs or whether they formed new modules with unique expression patterns. Of the 221 miRNAs differentially expressed during reprogramming with an FDR of 1% (FDR1 dataset), and 325 miRNAs differentially expressed at an FDR of 5% (FDR5 dataset), 66% and 69%, respectively, are miRNAs that were previously unknown to be differentially expressed during reprogramming.

**Figure 3:**
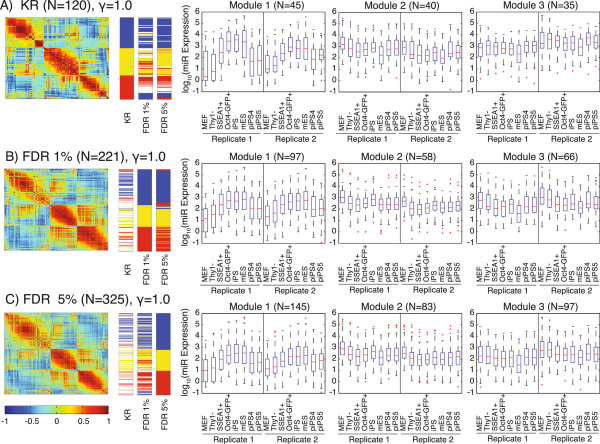
**Community structure performed at gamma = 1.** For the **(A)** KR, **(B)** FDR1 and **(C)** FDR5 datasets, the heatmap of the correlation matrix is on the left; each row and column represents a single miRNA. Color bars indicate the submodule assignment in each dataset of every miRNA in the heatmap. Color bar segments are labeled with the module they represent. Module 1 is blue, 2 is yellow and 3 is red. Not all miRNAs in each dataset are present in the other two; miRNAs that are absent are white in the color bar. Whisker plots show the log_10_(expression) of the miRNAs in the submodule. See also Figures S4, S5 and S6 in Additional file 1.

Interestingly, the addition of the large number of poorly annotated miRNAs to the network did not greatly alter the modular structure (Figure 3; Tables S4 and S5 in Additional file 1). The patterns of expression in all three datasets partitioned into similar modules of co-regulated miRNAs (Figure 3; Figure S4 and Table S5 in Additional file 1). With the exception of a very few miRNAs, the large numbers of miRNAs with unknown roles in reprogramming in the FDR1 or the FDR5 datasets did not create novel modules compared to the KR dataset, but were included in those modules created from miRNAs with known involvement in reprogramming. To detect finer-scale modular structure, the structural resolution parameter γ was increased from the standard value of 1 to 2.5 (Figure S5 in Additional file 1). The major effect of the increase in γ was a split of module 2 at γ = 1 into at least two modules, while modules 1 and 3 remained largely intact (Figure S5 in Additional file 1). At γ values above 2.5, additional modules formed were largely composed of single miRNAs (data not shown).

**Figure 4:**
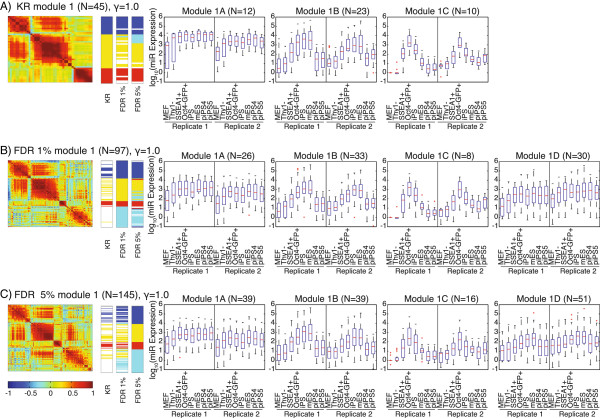
**Community structure of miRNAs found in module 1 of the representative partition of the original modular decomposition performed at gamma = 1.** For the **(A)** KR, **(B)** FDR1 and **(C)** FDR5 datasets, the heatmap of the correlation matrix is shown on the left. Each row and column represents a single miRNA. Color bars indicate the submodule assignment in each dataset of every miRNA in the heatmap to the left. Submodule 1 is blue, 2 is yellow, 3 is red and 4 is cyan. Not all miRNAs in each dataset are present in the other two; miRNAs that are absent are white in the color bar. Whisker plots show the log_10_(expression) of the miRNAs in the submodule. See also Figure S4 in Additional file 1.

**Figure 5:**
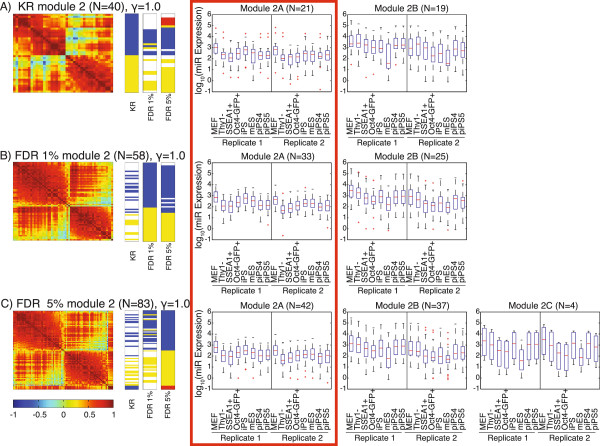
**Community structure of miRNAs found in module 2 of the representative partition of the original modular decomposition performed at gamma = 1.** For the **(A)** KR, **(B)** FDR1 and **(C)** FDR5 datasets, the heatmap of the correlation matrix is shown on the left. Each row and column represents a single miRNA. Color bars indicate the submodule assignment in each dataset of every miRNA in the heatmap to the left. Submodule 1 is blue, 2 is yellow and 3 is red. Not all miRNAs in each dataset are present in the other two; miRNAs that are absent are white in the color bar. Whisker plots show the log_10_(expression) of the miRNAs in the submodule. The red rectangle highlights module 2A, dominated by *Dlk1*-*Dio3* miRNAs; 15 of 21 in the KR dataset, 31 of 33 in the FDR1 dataset and 39 of 42 in the FDR5 dataset are from the *Dlk1*-*Dio3* region. See also Figure S4 in Additional file 1.

The substructure within modules was investigated by an independent analysis of the subset of miRNAs within each module detected at the default resolution of γ = 1. From this analysis multiple submodules arose (Figures 4 and 5; Figures S4 and S6 in Additional file 1). The pattern of expression in submodule 1A is characteristic of stem cell miRNAs upregulated early in reprogramming. Among them are miRNAs expressed in piPSC as well as iPSC and mES cell lines, including some members of the 17 ~ 92 and 106b ~ 25 clusters and MET miRNAs (Figure 4). Submodule 1B generally consisted of miRNAs that were upregulated in the middle stages of reprogramming (mainly Thy1- to SSEA+), including members of the 290 ~ 295 cluster and MET miRNAs. miRNAs in this module had high expression in stem cells, but low expression in piPSCs. Therefore, this module appears to identify miRNAs that change relatively early to ensure successful re-programming (Additional file 1). Submodule 1C had an expression pattern of transient upregulation during reprogramming. miRNAs in this submodule included the 302b ~ 367 cluster, and miR-489-3p. The 302b ~ 367 cluster is active in regulating the cell cycle of stem cells [44] and the expression of these miRNAs alone can reprogram fibroblasts to stem cells [45]. A fourth submodule, 1D, was only found in the FDR1 and FDR5 datasets, and had a trend of increasing expression during reprogramming. Members of this submodule included those from reprogramming- and pluripotency-associated miRNA clusters 17 ~ 92a, 106a ~ 363, and 106b ~ 25.

The network analysis of module 2 miRNAs resulted in two submodules in the KR and FDR1 datasets, and three submodules in the FDR5 dataset (Figure 5). Submodule 2A is characterized by a sharp decrease in expression between the MEF and Thy1- samples, and all 11 miRNAs in this submodule are *Dlk1*-*Dio3* miRNAs, corresponding well to the edgeR analysis, where *Dlk1*-*Dio3* miRNAs made up 81% of miRNAs (66/81) with a significant decrease in expression between these two stages (Figure 5, red rectangle). Submodule 2B is composed of miRNAs that decrease in expression throughout reprogramming, including let-7 miRNAs (let-7b,e,i-5p). The final submodule, 2C, is found only in the FDR5 dataset and is composed of only four miRNAs (miR-199a-5p, miR-145-5p, miR-155-5p, miR-143-5p), including two differentiation-associated miRNAs (miR-145-5p, miR-155-5p), and tends to have significant decreases in expression early in reprogramming. In the γ = 1 network analysis, module 3 showed little overall variation in miRNA expression and the expression patterns of several submodules were inconsistent between replicates (Figure S6 in Additional file 1).

### Experimentally testing the role of *Dlk1*-*Dio3* miRNAs in reprogramming

**Figure 6:**
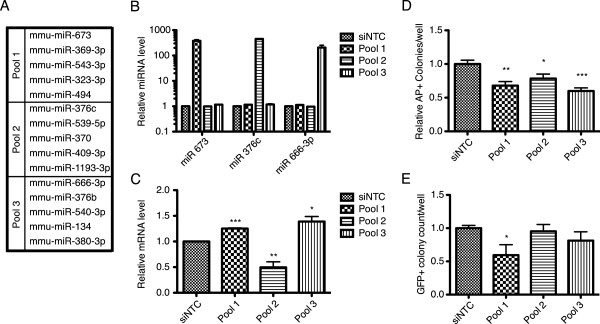
**Effect of *Dlk1-Dio3 *miRNAs on reprogramming. (A)** Three pools of five downregulated miRNAs located within the *Dlk1*-*Dio3* gene cluster. **(B)** Quantitative PCR of representative miRNAs from pools 1 to 3 to assess transfection efficiency. **(C)** Quantitative PCR analysis of Thy1 expression 5 days after OSKM infection and miRNA pool transfection. **(D)** Relative number of AP + colonies per well 12 days after OSKM infection and miRNA pool transfection. **(E)** Relative number of Oct4-GFP+ colonies per well 14 days after OSKM infection and miRNA pool transfection. siNTC, si no-template control, *p < 0.05, **p < 0.01, ***p < 0.001, determined by two-tailed Student’s t test.

To validate the results of the deep sequencing patterns and determine whether the downregulation of 66 *Dlk1*-*Dio3* miRNAs at the MEF to Thy1- transition affected reprogramming efficiency, 15 *Dlk1*-*Dio3* miRNAs were tested in cells. Given the poor functional knowledge of many of the miRNAs in this region, we used TargetScan to identify miRNAs with potential roles in reprogramming. TargetScan [46] data on the *Dlk1*-*Dio3* miRNAs were analyzed to identify putative mRNA targets that play important roles in reprogramming, embryonic stem cell biology, cell cycle and gene expression. The 15 miRNAs with the most predicted targets associated with these functions were selected for further investigation (Figure 6A). Many of these chosen miRNAs, such as miR-673-5p, miR-369-3p and miR-1193-3p, have previously been linked to embryonic stem cells and newborn mouse tissues [47-49].

To show the functional relevance of these 15 selected miRNAs in iPSC induction, iPSCs were generated by transfecting three pools of 5 miRNA mimics each into Oct4-GFP MEFs on the same day as four-factor transduction and then again 5 days post-infection. Mimics rather than inhibitors were transfected because the miRNAs are already significantly downregulated during four-factor induction and further inhibition may have a negligible effect on the targets. Quantitative RT-PCR analysis showed robust and highly specific miRNA expression three days post-transfection (Figure 6B). While transfection levels measured by quantitative PCR were high (approximately 500-fold), the actual active dose within cells is much lower (on the order of 1 to 2%) since not all copies reach the cytoplasm, and not all bind to Ago2 [50].

Pool 1 miRNAs showed a clear effect on reducing the efficiency of transition to Thy1-, to AP+, and to Oct4-GFP+ (Figure 6). Pool 3 also impaired Thy1 downregulation while pool 2 induced an unexplained decrease in Thy1 mRNA levels (Figure 6C). At 12 to 16 days after induction, all pools of mimics showed a decrease in the mES cell marker AP and, in the case of pool 1, a decrease in Oct4-GFP (Figure 6E). While transfection of miRNAs could potentially result in non-specific interactions that could cause a reduction in reprogramming efficiency, the different response of the three pools suggests that this is not the case here. Overall, our results suggest that at least a subset of the downregulated miRNAs from the *Dlk1*-*Dio3* locus play a role in the transition from MEF to Thy1- and contribute to iPSC generation.

## Discussion

The fine structure changes observed by deep sequencing throughout reprogramming reveal a massive re-setting of the miRNA profile in which functionally related miRNAs operate in co-expression networks. Among the observed expression patterns are sets of miRNAs that gradually increase or decrease across reprogramming and other patterns that show sharper, more transient spikes in expression. Constitutive miRNAs are rare. To reveal the details of these complex changes we have analyzed reprogramming intermediates with two statistical techniques - edgeR to detect differentially expressed miRNAs and community detection to identify putative co-regulatory modules. These statistical techniques applied to deep sequencing data provide unprecedented detail concerning stage by stage miRNA expression changes during reprogramming.

Sampling at each reprogramming transition simply delineates the constellation of miRNA changes across a bulk sample associated with achieving the next step in reprogramming, and not those changes sufficient to achieve pluripotency. The barrier across the initial MEF to Thy1- transition is not high, as indicated by the large number of cells that become Thy1- after OSKM (Figure 1A). The barrier to each subsequent stage coincides with a reduced number of differentially expressed miRNAs, a larger fraction of which undergo large fold changes.

miRNAs from the imprinted *Dlk1*-*Dio3* region on chromosome 12qF1 dominated the miRNAs downregulated at this transition. In MEF reprogramming the long non-coding RNA *Meg3* (also known as *Gtl2*), which is expressed on the same imprinted maternal strand as the *Dlk1*-*Dio3* miRNAs, was also downregulated by the time reprogrammed MEFs became SSEA1+, but before the upregulation of pluripotency-associated *Nanog*[51]. Our data further localize the downregulation of the maternally expressed, non-coding RNAs at this locus to the MEF to Thy1- transition. While miRNAs at the *Dlk1*-*Dio3* locus underwent a broad downregulation (66 of 85 miRNAs) of modest fold change at the MEF to Thy1- transition, only five were upregulated at either of the later stage-to-stage transitions. Nevertheless, comparing the initial downregulated *Dlk1-Dio3* miRNA levels to the levels of these miRNAs in Oct4-GFP+ cells, a broader trend toward gradual re-expression was observed, with only 35 miRNAs still downregulated compared to their starting levels in MEFs.

Interestingly, a re-analysis of the microarray data in Polo *et al.*[11] also suggested a decrease in expression of *Dlk1-Dio3* region miRNAs during reprogramming of MEFs to iPSCs (Figure S3 in Additional file 1). Polo *et al.*[11] did not sample a Thy1- intermediate, and did not detect downregulation of *Dlk1-Dio3* miRNAs in their samples. In our re-analysis, we could detect downregulation of the *Dlk1-Dio3* miRNAs when comparing our MEF and SSEA1+ samples (our first and third time points, comparable to Polo *et al.*’s first and second time points), although it was not as strong across this wider interval. In our data between MEF and SSEA1+ stages, 58 of the 66 miRNAs downregulated at the MEF to Thy1- transition, as well as four additional *Dlk1-Dio3* miRNAs, were significantly downregulated. Reanalysis of Polo *et al*.’s data showed that 23 of 32 *Dlk1-Dio3* miRNAs in their single replicate have a negative log_2_ fold change in the transition from MEF to SSEA1+ (Figure S3B in Additional file 1; compared to 100 of 220 non-*Dlk1-Dio3* miRNAs).

The pattern of abrupt downregulation and gradual upregulation was almost exclusively a feature of miRNAs in the *Dlk1*-*Dio3* cluster and readily observable in the analysis of co-regulatory modules (Figure 5). This suggests one of two possibilities. First, that the majority of cells were downregulated at the *Dlk1-Dio3* locus during the MEF to Thy1- transition, but some maintained higher, MEF-level expression at this locus, leading to a net modest downregulation of these miRNAs. As reprogramming progressed, cells that downregulated *Dlk1-Dio3* miRNAs were reprogrammed less efficiently and therefore made up a decreasing proportion of the total cells, thereby minimizing the role of the miRNA downregulation. Second, that nearly all cells in the ensemble population undergo a transient downregulation of *Dlk1-Dio3* miRNAs early in reprogramming, with the expression of these miRNAs increasing as reprogramming progresses. This alternative is supported by overexpression of miRNA mimics from this locus. Some of these miRNAs decreased reprogramming efficiency. In particular, pool 1 mimics increased Thy1 expression, while decreasing the proportion of AP + and Oct4-GFP+ cells, and therefore reduced reprogramming efficiency. No pool of miRNA mimics increased reprogramming efficiency. Although single cell analyses will likely reveal additional details, the downregulation of *Dlk1*-*Dio3* miRNAs at the earliest stage of reprogramming, particularly those in pool 1, appear to be beneficial for reprogramming efficiency, although not essential for reprogramming success (Figure 6).

A possible function of *Dlk1-Dio3* miRNAs that would increase reprogramming efficiency may be their role in the epithelial-to-mesenchymal transition (EMT). A recent study [52] demonstrated that reprogramming efficiency in MEFs and several other cell types is improved by inducing EMT at the initial stage of reprogramming before MET occurs, via either TGFβ or the sequential introduction of reprogramming factors. They suggest that since MEFs are usually not uniformly mesenchymal (as they are generally derived from day 13.5 embryos, as is the case here), an initial EMT serves to convert MEFs to a more consistently mesenchymal state before MET initiation [52]. Seven *Dlk1-Dio3* miRNAs target *Twist1* and other EMT-associated genes, and their downregulation has been shown to induce EMT [53]. Of these seven, six were downregulated at the MEF to Thy1- transition in our dataset. The seventh, which was not significantly downregulated, had low abundance. Interestingly, the *Dlk1-Dio3* miRNAs miR-369-5p and -3p are able to reprogram MEFs when transfected with miR-200c-3p and miR-302a/b/c/d-3p, albeit at lower efficiency than OSKM [54]. Although the specific role of miR-369 has not been shown experimentally, it has been predicted to target the TGFβ pathway, indicating it may contribute to reprogramming by promoting MET at the Thy1- to SSEA1+ transition [54]. We speculate that early downregulation of these MET miRNAs may help induce an initial EMT at the MEF to Thy1- transition, as in Liu *et al.*[52], though via a different mechanism.

miR-369-3p and two of the seven miRNAs recognized as affecting MET by Haga and Phinney [53], miR-543-3p and miR-494-3p, were in pool one of the miRNA mimics, whose overexpression resulted in a decrease in both AP+ and Oct4-GFP+ cells (Figure 6). The overexpression of these mimics may, therefore, have reduced reprogramming efficiency by preventing an early EMT. Interestingly, pools 2 and 3 contained only one and zero, respectively, of the EMT-targeting miRNAs [53] and had less effect on reprogramming efficiency.

The patterns of miRNA expression described here are more complex than the 'two waves' described by Polo *et al.*[11] using microarrays. The greater dynamic range of expression levels by deep sequencing revealed patterns of regulation readily captured in the network analysis. The strong modularity observed in the networks of the large datasets and the complexity within the fine structure of the modules suggest the existence of network motifs that generate considerably more complex expression patterns than 'two waves.'

**Figure 7:**
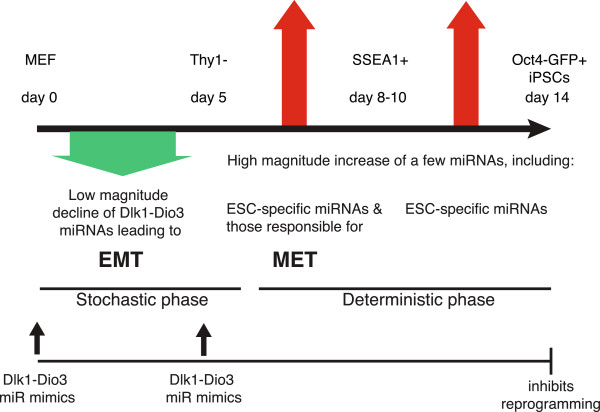
**Model of reprogramming stages.** Initially the cell population undergoes relatively uniform small fold changes in miRNA expression or experiences heterogeneous expression within the population. The downregulation of *Dlk1-Dio3* miRNAs at this stage may set up an initial EMT to prepare the sample for subsequent MET. The initial stochasticity is followed by a hierarchical phase where only a few miRNAs are differentially expressed, but at high fold changes. ESC, embryonic stem cell.

Reprogramming factors are believed to initiate a sequence of probabilistic events that generate a small and unpredictable fraction of iPSCs [55,56]. Buganim *et al.*[17] investigated patterns of gene expression in single cells undergoing reprogramming and found variation in the order of gene expression among sister cells of initial colonies early in the process, but a clear sequence of gene expression once core stem cell circuitry was activated. These data led to Buganim *et al.*’s [17] hypothesis that stochastic gene expression changes early in reprogramming are followed by a deterministic 'hierarchical' gene expression pattern responsible for the activation of the endogenous pluripotency circuitry. The sweeping small fold changes at the earliest stage of reprogramming may arise from heterogeneity within the population of cells or may represent sample-wide small fold changes. In either case, such phenomena are subject to greater stochastic behavior because noise in the system will have a greater proportional impact. Coinciding with the shift from a stochastic phase to a more hierarchical phase, the overall miRNA expression pattern shifts to larger magnitude changes among a smaller number of deterministic miRNAs as reprogramming progresses (Figure 7).

## Conclusions

During reprogramming, the miRNA profile of cells undergoes extensive changes. We identified key clusters of putatively co-regulated miRNAs, identifying patterns of coexpression during the reprogramming process. *Dlk1-Dio3* miRNAs were downregulated at the earliest stage of reprogramming, and functional experiments suggest this downregulation may improve reprogramming efficiency. While many miRNAs experienced small changes in expression at the earliest stages of reprogramming, only a few miRNAs experienced large changes in expression later in reprogramming, coinciding with the previously proposed shift from a stochastic to a hierarchical phase of reprogramming.

## Materials and methods

### Cell isolation

We seeded 5 × 10^5^ Oct4-GFP MEFs (derived from Jackson Lab stock number 008214) in 10-cm dishes and one day later transduced these with 10 ml 4 F virus supernatant (encoding Oct4, Sox2, Klf4, and cMyc; see Additional file 1 for additional details) [57]. The next day, the culture medium was replaced with fresh MEF medium, and 3 days later the medium was changed to embryonic stem culture medium. Intermediate stages of reprogramming were purified by FACS (Additional file 1). Several partially programmed iPSCs (piPSC or pre-iPSCs) with similar morphology and proliferative capacity as embryonic stem cells were derived, but remained reliant on transgene expression, and were GFP-negative, indicating they had not yet initiated the endogenous embryonic stem cell self-renewal regulation network. Libraries were prepared from these fractions and deep sequenced (Additional file 1).

### Mapping

Reads were mapped using the SOLiD Small RNA Analysis Tool (Applied Biosystems, Foster City, CA, USA), first to miRBase v.16 mouse miRNAs and then to the mouse genome. Reads were mapped uniquely allowing 0 or 1 color space mismatch. miRNA 5p and 3p arms were considered separately, and multi-copy miRNAs with identical mature miRNA sequences in several genomic loci were collapsed into single miRNAs (for example, miR-9-1-5p, miR-9-2-5p and miR-9-3-5p were combined into miR-9-5p). The first replicate was prepared with the Small RNA Expression Kit (Applied Biosystems, part number 4399434), which yielded 14.9 to 17.4 million reads per sample. The second replicate used a more efficient library preparation (SOLiD Total RNA-Seq Kit, Applied Biosystems, part number 4445374), to increase the read number (24.4 to 29.5 million reads per sample). An average of 3.8 million reads (1.9 and 5.6 million in each replicate, respectively) matched known mouse miRNA precursors from miRBase and an additional average of 4.5 million (2.3 and 6.6 million reads in each replicate, respectively) matched exons, introns and intergenic regions in the mouse genome. Reads for all samples (MEFs, Thy1-, SSEA1+, Oct4-GFP+, iPSCs, mES cells, piPS4, piPS5, both replicates) were previously combined into a single bulk sample and analyzed for isomiRs and isomoRs [20], but not for expression patterns among the reprogramming samples. The data have been made publicly available through the NCBI Sequence Read Archive database under accession numbers SRP010169 and SRP 010170. The present study is the first to compare individual samples at discrete time points during reprogramming.

### miRNA expression analysis

Data were trimmed mean of M (TMM) normalized using the BioConductor package edgeR v.3.2.1 [22,58]. PCA was carried out in R [59] on the most variant top half (290) of miRNAs, using TMM-normalized data centered and scaled across each miRNA. PCA revealed systematic differences in expression between the biological replicates due to the different kits used in library preparation. Differential expression analyses were therefore carried out in edgeR v.3.2.1 [22,58] using a multifactor model to investigate differences among treatments while taking into account differences between the two replicates [60]. edgeR was used to investigate (a) which miRNAs showed differential expression over the whole course of reprogramming (MEF → Thy1-, MEF → SSEA1+, MEF → Oct4-GFP+), and over each transition between consecutive stages (MEF → Thy1-, Thy1- → SSEA1+, and SSEA1+ → Oct4-GFP+), and (b) which miRNAs were differentially expressed in the piPSC lines compared to MEFs, stem cells (Oct4-GFP+, iPSCs and mES cells) and/or intermediate reprogramming stages (Thy1-, SSEA1+). For edgeR analyses, data were filtered to remove all miRNAs with fewer than 4 cpm in at least one library in each replicate, and all comparisons utilized a Benjamini-Hochberg correction for multiple tests. IsomiR analysis was conducted as previously described [20] with some modification (Additional file 1). In a previous study [20] investigating miRNA variation in a bulk, combined sample of the samples presented here, as well as hippocampal samples, we found the incidence of miRNA editing to be low (<6%); therefore, we did not pursue miRNA editing further in this study. A network analysis was conducted [61] (Additional file 1) with the standard structural resolution parameter (γ) of 1.0.

### Effect of *Dlk1*-*Dio3* miRNAs on reprogramming

EdgeR analysis of the deep sequencing data indicated that 66 of the 81 miRNAs that were downregulated from MEF to Thy1- were in the *Dlk1*-*Dio3* region. TargetScan was able to predict target genes of 41 downregulated *Dlk1*-*Dio3* miRNAs. To reduce the effects of TargetScan false positives, subsequent pathway analysis using IPA (Ingenuity Systems, Redwood City, CA, USA) [62] focused on the 1,296 genes that were targeted by at least 2 different *Dlk1*-*Dio3* miRNAs. These genes were filtered by their annotated function and used to identify15 *Dlk1*-*Dio3* miRNAs that regulate reprogramming genes for experimental validation.

Oct4-GFP MEFs (derived from Jackson Lab, stock number 008214 at E13.5) were cultured in DMEM with 10% fetal bovine serum, glutamine and nonessential amino acids (NEAA). Retroviruses for reprogramming were produced in the same way as the original reprogramming experiments (Additional file 1). MEFs were transfected with small interfering RNA (Dharmacon, Lafayette, CO USA) using Lipofectamine 2000 reagent (Invitrogen**,** Carlsbad, CA USA) 3 hours prior to four-factor transduction and again 5 days post-transduction. For iPSC induction, Oct4-GFP MEFs were seeded in gelatin coated 12-well plates and transduced with the combined virus plus 6 μg/ml polybrene the next day. The viral supernatant was replaced with fresh MEF medium the following day. On day 3 post-transduction, the culture medium was changed to mES cell medium consisting of DMEM with 15% fetal bovine serum (Hyclone, Logan, UT USA) plus LIF (Millipore, Billerica, MA USA), thioglycerol, glutamine and NEAA. GFP + colonies were counted 2 weeks post-transduction.

Total RNAs were extracted using Trizol (Invitrogen). For mRNA assays, 1 mg was used for reverse transcription using iScript (Bio-Rad, Hercules, CA USA) followed by quantitative PCR using a Roche LightCycler480 II (Roche, Basel, Switzerland) and SYBR Green (Bio-Rad). miRNAs were assayed using approximately 1.5 to 3 mg of total RNA for reverse transcription and quantitative PCR using the QuantiMir Kit (System BioSciences, RA420A-1, Mountain View, CA USA).

## Abbreviations

AP: alkaline phosphatase; cpm: counts per million; DMEM: Dulbecco's modified Eagle medium; EMT: epithelial-to-mesenchymal transition; FACS: fluorescence activated cell sorting; FDR: false discovery rate; GFP, green fluorescent protein; iPSC: induced pluripotent stem cell; MEF: mouse embryonic fibroblast; mES: mouse embryonic stem; MET: mesenchymal-to-epithelial transition; miRNA: microRNA; OSKM: Oct4 Sox2 Klf4 c-Myc; PCA: principal component analysis; PCR: polymerase chain reaction; piPSC: partial induced pluripotent stem cell; TGF: transforming growth factor.

## Competing interests

KSK owns stock in and serves on the board of Minerva Biotechnologies Corporation. The remaining authors declare no conflicts of interest.

## Authors' contributions

CMH, ZL, TMR and KSK: concept and design, data analysis and interpretation, manuscript writing. CMH carried out all analyses of the RNAseq data. JD, KYC, JL: stem cell reprogramming data collection, data assembly and analysis of these data. MLA prepared the libraries and carried out SOLiD sequencing. HZ mapped and quantified the RNAseq data. DSB provided Matlab code and advised on network analyses of the SOLiD sequencing data. All authors read and approved the final manuscript.

## Supplementary Material

Additional file 1 **Supplemental Methods, Results, Figures S1 to S6, and Tables S4 and S5.** Supplemental Methods include additional information about retrovirus and transduction, isolation of intermediate reprogramming states, library preparation, SOLiD sequencing, mapping, principal component analysis, microarray re-analysis, isomiR analysis, and network analysis. Supplemental Results include detailed results from network analyses. **Figure S1.** experimental verification of Thy1- cells. **Figure S2.** first four principal components of the principal component analysis. **Figure S3.** isomiR variation for miR-485-3p and microarray re-analysis. **Figure S4.** average correlation and partition similarity (z-score) for network analysis. **Figure S5.** fine-scale community structure uncovered at a larger value of the community resolution parameter (gamma = 2.5). **Figure S6.** community structure of miRNAs found in module 3 of the representative partition of the original modular decomposition performed at gamma = 1. **Table S4.** summary statistics for community structure analyses for all datasets. **Table S5.** statistical comparisons between network partitions for all analyses [63-82]. Click here for file

Additional file 2: Table S1 EdgeR log_2_ fold changes between MEFs, intermediate reprogramming stages, stem cells (labeled SC; Oct4-GFP+ newly reprogrammed cells, iPSC, and mES cell lines combined), and piPSCs. Values are significant at an FDR of 5%, bold values are significant with an FDR of 1%. Click here for file

Additional file 3: Table S2 EdgeR log_2_ fold changes in reprogramming. Values are significant at an FDR of 5%, bold values are significant with an FDR of 1%. log_2_ cpm is log_2_ of counts per million. Click here for file

Additional file 4: Table S3 miRNAs that are known from the literature to be differentially expressed between MEFs and iPSCs or embryonic stem cells, and/or to enhance reprogramming. The five miRNAs in italics did not meet the basic abundance requirements for edgeR analysis and were not considered in further analyses, including the network analysis. Click here for file
