# Supplementary material for: Staged miRNA re-regulation patterns during reprogramming
Source: Genome Biol. 2013 Dec 31;14(12):R149. doi: 10.1186/gb-2013-14-12-r149 (PMC4053856; doi:10.1186/gb-2013-14-12-r149)
Supplement: Additional file 1 — Supplemental Methods, Results, Figures S1 to S6, and Tables S4 and S5. Supplemental Methods include additional information about retrovirus and transduction, isolation of intermediate reprogramming states, library preparation, SOLiD sequencing, mapping, principal component analysis, microarray re-analysis, isomiR analysis, and network analysis. Supplemental Results include detailed results from network analyses. Figure S1. experimental verification of Thy1- cells. Figure S2. first four principal components of the principal component analysis. Figure S3. isomiR variation for miR-485-3p and microarray re-analysis. Figure S4. average correlation and partition similarity (z-score) for network analysis. Figure S5. fine-scale community structure uncovered at a larger value of the community resolution parameter (gamma = 2.5). Figure S6. community structure of miRNAs found in module 3 of the representative partition of the original modular decomposition performed at gamma = 1. Table S4. summary statistics for community structure analyses for all datasets. Table S5. statistical comparisons between network partitions for all analyses [63-82]. [file gb-2013-14-12-r149-S1.docx]

**Additional file 1 – Supplemental Methods, Results, Figures, and Table S4**

**Supplemental Methods**

**Retrovirus production and transduction**

5.5x10^6^ PLA-E cells were seeded and the next day transfected with 9 µg of pMX vectors (encoding Oct4, Sox2, Klf4, and cMyc). Medium was refreshed one day after transfection and virus-containing supernatants were collected 24 h later. The ‘four factor’ (4F) virus supernatants were combined and centrifuged at 4000 rpm for 5 min to remove cell debris, filtered through a 0.45 µm syringe filter (Millipore, Cat.SLHV33RS), and supplemented with 4 µg/ml Polybrene (Millipore, Cat.TR-1003-G).

**Isolation of intermediate states during reprogramming**

Oct4-GFP MEFs were derived from mice carrying an IRES–EGFP fusion cassette downstream of the stop codon of *Pou5f1/Oct4* [1, 2] (Jackson Lab, Stock #008214), and transfected with 10 ml ‘four factor’ (4F) virus supernatant (encoding Oct4, Sox2, Klf4, and cMyc).

The starting population of MEFs become GFP+ when *Pou5f1/Oct4* is endogenously activated and they are fully reprogrammed to an ES-like stage. During reprogramming, Thy1+ MEFs first down-regulate expression of MEF-specific markers and become Thy1–, then SSEA1 expression is induced in a small percentage of cells. These cells are capable of becoming GFP+ once fully reprogrammed [3, 4]. Initially, 85% of MEFs were positive for Thy1 (Figure 1A), and five days after transduction with the OSKM viruses ~45% of cells became Thy1– (Figure 1A), consistent with the previous report [3]. At day 9 to 10 post-transduction, 5.5% of the cells were SSEA1-positive (Figure 1B), and by day 14 ~3.7% of the cells reached the fully reprogrammed state, as judged by GFP expression (Figure 1C).

For purification of the Thy1– population, cells were harvested from day five post-transduction cultures, trypsinized into single cell suspensions and washed once in PBS containing 0.5% BSA (w/v). Approximately 1x10^7^ cells were stained with ~80 μl PE-conjugated anti-mouse Thy1 antibody (eBioscience, San Diego, CA; Cat.12-0902-82) in 1 ml of 0.5% BSA/PBS for 30 min on ice and then FACS-sorted (Fig. 1A). As positive controls for FACS-sorting, uninfected Oct4-GFP MEFs were stained in an identical manner. Unstained MEFs were used as staining controls. To verify the functional integrity of the sorted cells and the progression of 4F reprogramming, 4x10^4^ Thy1– and Thy1+ cells were collected and reseeded on irradiated MEF feeder plates until day 14. The cells were then cultured in mES medium for 14 days to evaluate their ability to form GFP^+^ iPSC colonies; Thy1– cells formed at least 10-fold more cells with iPSC-generating potential than Thy1+ cells (Fig. S1A).

For purification of the SSEA1+ population, cells were harvested from culture on day 9 or 10 post-4F transduction. Cells were trypsinized into single cell suspensions and washed once with 0.5% BSA/PBS. Cells were stained as described above with APC-conjugated anti-SSEA1 (R&D Systems, Minneapolis, MN; Cat. FAB2155A), according to the manufacturer’s recommendations. For purification of the GFP+ population, cells were harvested into single cell suspensions and sorted without further staining.

To verify the progression of 4F reprogramming, 4x10^4^ Thy1– and Thy1+ cells were collected and reseeded on irradiated MEF feeder plates until day 14. The cells were then cultured in mES medium for 14 days to evaluate their ability to form GFP^+^ iPSC colonies. Analysis of these cultures confirmed the presence of many alkaline phosphatase (AP)-positive colonies (indicative of ES cells) in the cultures originating from Thy1– cells, with at least 10-fold more cells with iPSC-generating potential in the Thy1– than in the Thy1+ population, based on quantification of AP+ and GFP+ colonies (Figure S1B, S1C). For the cell sorting analyses, wild type MEFs and established iPSCs were used as negative and positive control cells for marker expression. These data confirmed that we successfully purified an intermediate cell population from the reprogramming process.

**Library preparation and sequencing**

RNA was extracted from two replicates of the MEF to Thy1– to SSEA1+ to Oct4-GFP+ reprogramming samples, as well as an additional iPS line, mES line, and two partial iPS lines (piPS4 and piPS5). The first library was prepared using the SOLiD Small RNA Expression Kit (SREK, Part #4445374, Applied Biosystems) and sequenced with SOLiD version 3 chemistry. The second replicate was prepared after the discontinuation of the SREK kit, and was instead prepared with its successor, Total RNA-Seq kit (Part #4399434) and SOLiD version 4.

**Mapping**

The SOLiD Small RNA Analysis Tool (Applied Biosystems) was used to map the colorspace reads to miRbase version 16 mouse miRNAs and then to the mouse genome. Reads were mapped uniquely allowing 0 or 1 color space mismatch. With the dibase reading method used by the SOLiD, one color space mismatch is equivalent to no mismatches in base space. Several of the version 16 mouse miRNAs have since been identified as fragments of rRNA or tRNA, or otherwise not miRNAs and removed from miRbase, and reads mapping to these miRNAs were removed from the dataset. These 80 dead miRNAs were removed from the dataset prior to analysis: mir-1274a-5p, mir-1274a-3p, mir-1937a-5p, mir-1937a-3p, mir-1937b-1-5p, mir-1937b-1-3p, mir-1937b-2-5p, mir-1937b-2-3p, mir-1937b-3-5p, mir-1937b-3-3p, mir-1937b-4-5p, mir-1937b-4-3p, mir-1937b-5-5p, mir-1937b-5-3p, mir-1937c-5p, mir-1937c-3p, mir-1939-5p, mir-1939-3p, mir-1944-5p, mir-1944-3p, mir-1959-5p, mir-1959-3p, mir-2132-5p, mir-2132-3p, mir-2133-1-5p, mir-2133-1-3p, mir-2133-2-5p, mir-2133-2-3p, mir-2134-1-5p, mir-2134-1-3p, mir-2134-2-5p, mir-2134-2-3p, mir-2134-3-5p, mir-2134-3-3p, mir-2134-4-5p, mir-2134-4-3p, mir-2134-5-5p, mir-2134-5-3p, mir-2134-6-5p, mir-2134-6-3p, mir-2135-1-5p, mir-2135-1-3p, mir-2135-2-5p, mir-2135-2-3p, mir-2135-3-5p, mir-2135-3-3p, mir-2135-4-5p, mir-2135-4-3p, mir-2135-5-5p, mir-2135-5-3p, mir-2138-5p, mir-2138-3p, mir-2140-5p, mir-2140-3p, mir-2141-5p, mir-2141-3p, mir-2145-1-5p, mir-2145-1-3p, mir-2145-2-5p, mir-2145-2-3p, mir-2146-5p, mir-2146-3p, mir-220-5p, mir-220-3p, mir-297a-5-5p, mir-297a-5-3p, mir-297a-6-5p, mir-297a-6-3p, mir-464-5p, mir-464-3p, mir-469-5p, mir-469-3p, mir-689-1-5p, mir-689-1-3p, mir-689-2-5p, mir-689-2-3p, mir-715-5p, mir-715-3p, mir-744-5p, mir-744-3p.

**Principal Component Analysis**

To reduce the effects of invariant miRNAs, only the top half most variant miRNAs were used to construct the PCA.

**Microarray analysis**

To confirm the pattern of downregulation of Dlk1-Dio3 miRNAs early in reprogramming, we inspected the pattern of expression of these miRNAs in microarray data from [5]. Raw data were downloaded from the GEO database (<http://www.ncbi.nlm.nih.gov/geo/>, Accession GSE42475), and following Polo et al. [5], data were loess normalized, probes with more than 3 missing values across samples were excluded, remaining missing values were imputed, and samples were quantile normalized across arrays. We examined the log_2_ fold change (log_2_ FC) between the first two stages sampled by Polo et al. [5], MEF and SSEA1+/Thy1–. Although this does not correspond to our MEF to Thy1– comparison, Polo et al. [5] did not sample a Thy1– timepoint between the MEF and SSEA1+ stages, and 62 of the 85 Dlk1-Dio3 miRNAs in our dataset were significantly downregulated between MEF and SSEA1+ samples (58 of these were among the 66 downregulated at the MEF to Thy1– transition). Since the Polo et al. [5] consisted of only a single replicate, we did not attempt statistics, simply looked at the log_2_FC of between the samples of Dlk1-Dio3 miRNAs compared to all miRNAs.

**Isomir analysis**

By deep-sequencing, variation in the exact start and end sites of mature miRNAs can be detected [1]. Zhou et al. [6] found variation among isomirs in an analysis of all samples in our study combined, so to investigate this further, we assessed whether any miRNAs experienced changes in their dominant isomir(s) through reprogramming. Given the proximity of the seed sequence to the 5’ end of miRNAs, variation in the 5’ end has far more potential to alter the targets of a miRNA than variation at the 3’ end. Following Zhou et al. [6], we focused on the 5’ end, and calculated the weighted average size of nucleotide variation, (WAZNV) as: WAZNV=Σ*p_i_d_i_,* where p_i_ is the frequency of isomir *i* among all isomirs detected, and d_i_ is the distance of isomir *i* from the mature miRNA as defined by miRBase (www.mirbase.org). (This is a slight departure from Zhou et al. [6], who defined d_i_ as the distance to the dominant isomir, regardless of whether it was the mature miRNA defined by miRBase.) To assess whether there was a difference in dominant isomir(s) in MEF vs. Oct4-GFP+ samples, WAZNV was calculated for these two endpoints of reprogramming for all miRNAs with at least 50 reads in these samples in both replicates. For each replicate, the difference in WAZNV between these two samples was calculated, and miRNAs were filtered for those with an absolute value of the mean of the difference >0.1. To retain only those with consistent patterns between replicates, these were further filtered to those with a correlation across reprogramming samples (MEF, Thy1–, SSEA1+ and Oct4-GFP+) >0.9 between replicates. Target sites of different isomirs were predicted using custom TargetScan (v. 5.2) [7].

**Network analysis**

Following Conaco et al. [8], a putative coregulation network of miRNAs was constructed for each of three datasets: i) 120 miRNAs known from the literature [9-25] to be functionally involved or differentially expressed during reprogramming (Known Reprogramming, or KR dataset, Table S3), ii) 221 miRNAs differentially expressed in our dataset with an FDR of 1% (FDR1 dataset) and iii) 325 miRNAs differentially expressed in our dataset with an FDR of 5% (FDR5 dataset). For the KR dataset, 125 miRNAs were identified from the literature, but five had read counts so low in one or both replicates that they did not meet the basic abundance requirements for edgeR analysis, and were not considered for the network analysis. Connections between miRNAs in the putative coregulation networks were weighted by the pairwise Pearson correlation of TMM-normalized expression values across all treatments. For each dataset, miRNA co-expression networks were constructed for both the full dataset (8 treatments in each of two replicates = 16 treatments) and just the reprogramming samples (4 treatments in each replicate = 8 treatments). The average correlation (R) for each dataset was calculated as the average of all pairwise Pearson correlations between each pair of miRNAs, and can be interpreted as a measure of the magnitude of putative co-regulation across the dataset.

We used a community detection approach [26, 27] that utilizes the Louvain method [28] to optimize a modularity quality function [29, 30]. Briefly, this method heuristically partitions the miRNAs into modules by maximizing the strength of expression correlations within modules.

Because the correlation matrices capturing co-expression patterns of miRNA pairs contain both positive and negative matrix elements, we employ an extension of the modularity quality function to networks with positive and negative links [31]. Specifically, We define the correlation matrix ***A*** and then define *w_ij­_^+^* to be an N×N matrix containing the positive elements of *A_ij_* and *w_ij_^−^* to be an N×N matrix containing only the negative elements of *A_ij_*. The quality function to be maximized is then given by the following equation:

$$Q_{\pm}=\frac{1}{2w^{+}+2w^{-}}\sum_{i} \sum_{j} \left[A_{ij}-\left(\gamma^{+}\frac{w_{i}^{+}w_{j}^{+}}{2w^{+}}-\gamma^{-}\frac{w_{i}^{-}w_{j}^{-}}{2w^{-}}\right)\right]\delta\left(g_{i},g_{j}\right)$$

where *g­_i_* is the community to which node *i* is assigned, *g_j_* is the community to which node *j* is assigned, γ^+^ and γ^-^ are resolution parameters, and the following equation applies [31, 32]:

$$w_{i}^{+}=\sum_{j} w_{ij}^{+},w_{i}^{-}=\sum_{j} w_{ij}^{-}.$$

The two free parameters in the optimization of the modularity quality function are then the resolution parameters [32, 33], which control the sensitivity of the algorithm to structure at larger or smaller scales in the network. For simplicity, we kept γ+ and γ- equal, (hereafter referred to as simply γ) and varied their values from 0.1 to 2 in intervals of 0.1 to examine their effect on the number and composition of putatively co-regulated modules detected.

Because the optimization of the modularity quality function is NP-hard we explore the near-degeneracy of solutions by performing 100 optimizations of the quality function for each value of γ. We calculate the average number of modules (S) and modularity (Q) over these optimizations. The average modularity, Q, can be interpreted as a measure of putative co-regulation of miRNAs within modules, with high values of Q indicating strong putative co-regulation of miRNAs within modules and low values of Q indicating weak putative co-regulation of miRNAs within modules.

A representative partition of the 100 optimizations was constructed as in Bassett et al. [34]. Briefly, a matrix T was created summarizing the number of times each pair of miRNAs in the dataset *i* and *j* were in the same module in each of the 100 optimizations. Each of the 100 optimizations was then randomly permuted, and a corresponding matrix T^r^ was constructed summarizing the number of times each pair of miRNAs was in the same module in each of the 100 randomly permuted optimizations. All values T*_ij_* which were lower than the corresponding values T_ij_^r^ were set to 0, and this modified T matrix was then subjected to the same community detection approach used to construct the original co-expression networks above to create a single, representative partition.

Community structuring was carried out at the standard resolution parameter of 1, and is discussed in detail in the main text. Increasing γ values generally resulted in a greater number of modules detected within a network. To investigate the effect of γ values greater than 1, we examined the network produced by a γ value of 2.5. The γ value of 2.5 was chosen because values greater than 2.5 resulted in increasing numbers of individual miRNAs being identified as separate, individual modules – a hallmark of fragmentation in the network. While the number of modules detected at γ=2.5 was increased in comparison to the number identified at γ=1, the majority of new modules were formed from small groups of miRNAs or single miRNAs splitting from the major modules detected at γ=1. The only consistent major subdivision of the existing modules was a splitting of a single module (module 2) into at least two submodules. Therefore, as a separate approach to examine the substructure within all of the modules detected at γ=1.0, each module was reanalyzed individually to detect submodularity.

To determine the significance of network community structure, two treatment-permuted null models were constructed for each dataset: i) for each miRNA, expression values were permuted over all treatments (randomly permuted across all treatments dataset), and ii) to account for potential differences between the two replicates even after normalization, for each miRNA, expression values were permuted within each replicate (randomly permuted within each replicate dataset). For both types of permutations, 1000 different permuted datasets were constructed, and average correlation (R) was calculated for each. One-sample, two-tailed t-tests were conducted comparing the 1000 R values to the mean R value from the true dataset. Similarly, the partitioning of each of the 1000 replicates was compared to the representative partition of the actual dataset via a z-score of the Rand coefficent [34, 35], which measures similarity between the networks. Z-scores were averaged over the replicates to generate a mean z-score for each permuted dataset. The mean expected z-score between independent networks is 0, with increasing z-scores for more-similar networks. When comparing networks of datasets with only partially overlapping sets of miRNAs, the z-score was calculated only for the miRNAs in common between the datasets. In the permuted datasets where the expression levels of each miRNA were randomly permuted across treatments within each replicate (within replicate, randomly permuted datasets), the permutation maintained the structure due to differences between replicates that is present in the real dataset. The mean z-scores between the actual dataset and within replicate, randomly permuted datasets were therefore elevated above zero, reflecting these preserved between replicate differences. For comparison, the mean z-score between each of the 100 optimizations of the true dataset compared to the representative partition was also calculated, to illustrate the z-score of partitions that share more than simply between replicate differences (Fig. S4).

**Supplemental Results**

**Modules of differentially expressed miRNAs are stable even without known reprogramming miRNAs, or when investigating only reprogramming samples**

There was high partition similarity (z-scores) between network module partitioning generated from the full FDR1 and FDR5 datasets and those partitions generated from their counterpart datasets where the miRNAs overlapping with the KR dataset had been removed (Table S5). Modules detected in both the FDR1 and FDR5 datasets were therefore largely the same even after the subset of miRNAs from the literature were removed. This indicates that the partitioning of these datasets into modules, and the similarity of this partitioning to the KR dataset, did not rely solely on the expression patterns of the miRNAs shared in common between the KR dataset and either the FDR1 or FDR5 datasets. Similarly, when only the reprogramming samples (MEF, Thy1–, SSEA1+ and Oct4-GFP+) were analyzed at γ=1, the results were quite similar to those for the entire dataset. The same number of modules were detected, and there was high partition similarity (z-scores) between the partitioning generated from the reprogramming-only sample datasets and that generated from the corresponding full datasets (Tables S4, S5). This suggests that the pattern of partitioning is robust and reflects differences in the expression patterns of various groups of miRNAs during reprogramming. For all datasets (Fig. S4), the average correlation was greater than expected by chance when compared to the average correlation of randomly permuted datasets, indicating greater than random co-expression among the miRNAs in the datasets. Similarly, the partition similarity (z-score) between network partitions of the randomly permuted datasets and the representative partition of the actual data was lower than between the representative partition and the underlying optimizations of the actual data (Extended Experimental Procedures, Fig. S4). The mean z-score for the representative partition of the actual data compared to the datasets created by randomly permutation across all treatments was 0 for all datasets and community resolution parameters, as expected between independent networks. The mean z-score for the representative partition of the actual data compared to the randomly permuted within each replicate datasets was greater than zero for some combinations of dataset and community resolution parameter, reflecting the between replicate structure shared between the representative partition and these permuted datasets. However, these z-scores were always lower than the mean z-score between the representative partition and the underlying optimizations of the actual data, demonstrating that the shared structure due to the differences between the two replicates was only a fraction of the total structure.

**Details of submodule structure when γ=2.5**

When the resolution parameter **γ** was increased to 2.5, the FDR1 dataset splintered into 9 modules, 5 of which had 10 or fewer miRNAs. The only consistent subdivision across all three datasets was that module 2 from the analysis at γ=1 was subdivided, though the extent of that subdivision varied greatly across the three datasets. In the KR dataset, modules 1 & 3 at γ=1 were largely identical to modules 1 and 2, respectively, at γ=2.5, while module 2 from γ=1 split into modules 3 and 4 at γ=2.5. In the FDR1 dataset, module 1 at γ=1 is identical to module 1 at γ=2.5 with the exception of 2 miRNAs, while module 3 at γ=1 largely corresponds to modules 2 and 5 at γ=2.5. Module 2 at γ=1, however, split into modules 3,4,6,7,8 and 9 (plus one miRNA contributed to module 1) when γ was increased to 2.5. The effects of increasing γ to 2.5 were not as severe for the FDR5 dataset, where modules 1, 2 and 3 at γ=1 roughly corresponded to modules 1, 3 and 2, respectively, at γ=2.5. The fourth module at γ=2.5 was composed of about half (7 miRNAs) from module 1 at γ=1 and half (8miRNAs) from module 2 at γ=1.

**Details of submodule structure in module 1**

The substructure of module 1 was investigated with a resolution parameter of γ=1 and yielded three modules in the KR dataset, and four in the FDR1 and FDR5 datasets. Members of submodule 1A included the majority of MET miRNAs (miR-200a-3p, miR-200b-3p, miR-429-3p, and miR-205-5p), two miRNAs of the 106b~25 cluster (miR-106b-5p and miR-93-5p), four miRNAs from the miR-17~92 cluster (miR-17-5p, miR-18a-5p, miR-20a-5p, and miR-92a-3p) and miR-183-5p. The 106b~25 and 106a~363 clusters are paralogs of the 17~92 cluster, are all activated by c-Myc and play a role in reprogramming [9, 36]. miR-93 and miR-106b, in particular, have been shown to increase reprogramming efficiency and promote MET by targeting p21 and TGFBR [9].

miRNAs in submodule 1B in all three datasets included most of the 290~295 cluster (miR-290-5p, miR-291a-3p, miR-291a-5p, miR-291b-3p, miR-291b-5p, miR-292-3p, miR-294-3p, miR-295-3p), two members of the 106a~363 cluster (**miR-106a-5p**, **miR-363-3p**) and two MET miRNAs (miR-141-3p, **miR-200c-3p**). Here and throughout, bold denotes miRNAs that were absent from the FDR1 dataset (i.e. not significant at an FDR of 1%), but in this submodule in both the KR and FDR5 datasets. The only other miRNAs in this submodule (either in all datasets or just KR and FDR5) were from the Dlk1-Dio3 cluster (**miR-495-3p**, miR-543-3p and **miR-758-3p**), which had the additional feature of early down-regulation in the MEF to Thy1– transition as described above, although miR-758-3p was one of the 19 miRNAs in this region not significantly downregulated at the MEF to Thy1– transition.

MiRNAs in submodule 1C included the 302b~367 cluster (miR-302a,b,c and d-3p, miR-367-3p and star sequences **miR-302a,c**,d-5p ), and miR-489-3p. MiRNAs in this submodule were upregulated in reprogramming, with their highest levels in the Oct4-GFP+ samples. Expression of these miRNAs was lower in the established iPS line and mES line, confirming Polo et al.’s [5] result, which found that miR-302a was transiently upregulated in Oct4-GFP+ cells only.

Of the 30 (FDR1) and 51 (FDR5) miRNAs in submodule 1D, 19 were members of this submodule in both the FDR1 and FDR5 datasets. Of these, six were mature (miR-19a-3p, 19b-3p) or star sequences (miR-20a-3p, mir-92a-1-5p, mir-92a-2-5p, mir-93-3p) from reprogramming- and pluripotency-associated miRNA clusters 17~92a, 106a~363, 106b~25, and one was a star sequence (miR-200b-5p) of a MET-associated miRNA.

**Supplemental Figures**

**
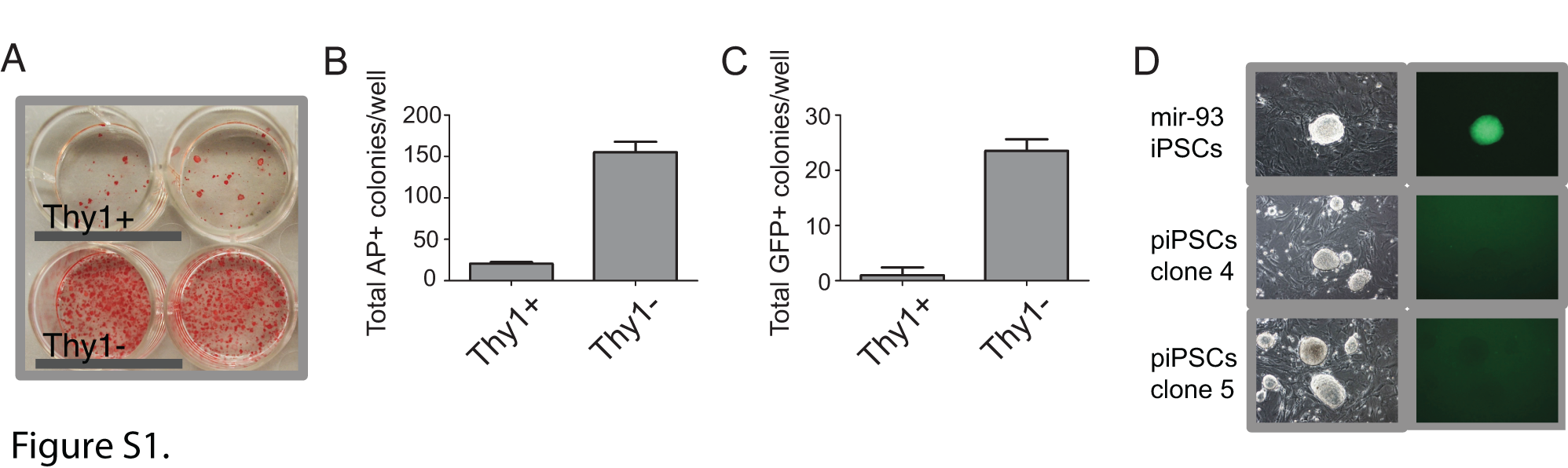
**

**Figure S1. Experimental verification of Thy1– cells**

A) The Thy1– population is enriched for potential iPSC cells. Thy1+ and Thy1– cells were sorted from OSKM-infected MEFs on 5 day of culture. An equal number of cells (4x10^4^) was reseeded onto irradiated feeder plates and cultured until day 13. Cells were then fixed and stained with an AP substrate. Red color indicates the AP+ colonies. B) Thy1– cells showed greater potential to form AP+ colonies. AP+ colonies were quantified on day 13 post-OSKM transduction. Graphs are mean and range of duplicate wells. C) The Thy1– population was enriched with iPSC-generating cells. Oct4-GFP+ colonies were quantified on day 13 post-OSKM transduction. Graphs are mean and range of duplicate wells. D) piPSCs were derived from OSKM-transduced MEFs. The cells have similar morphology and proliferation rate to mES cells and established iPSCs, but have not switched on expression of endogenous self-renewal markers such as *Oct4*, and thus remain GFP-negative.


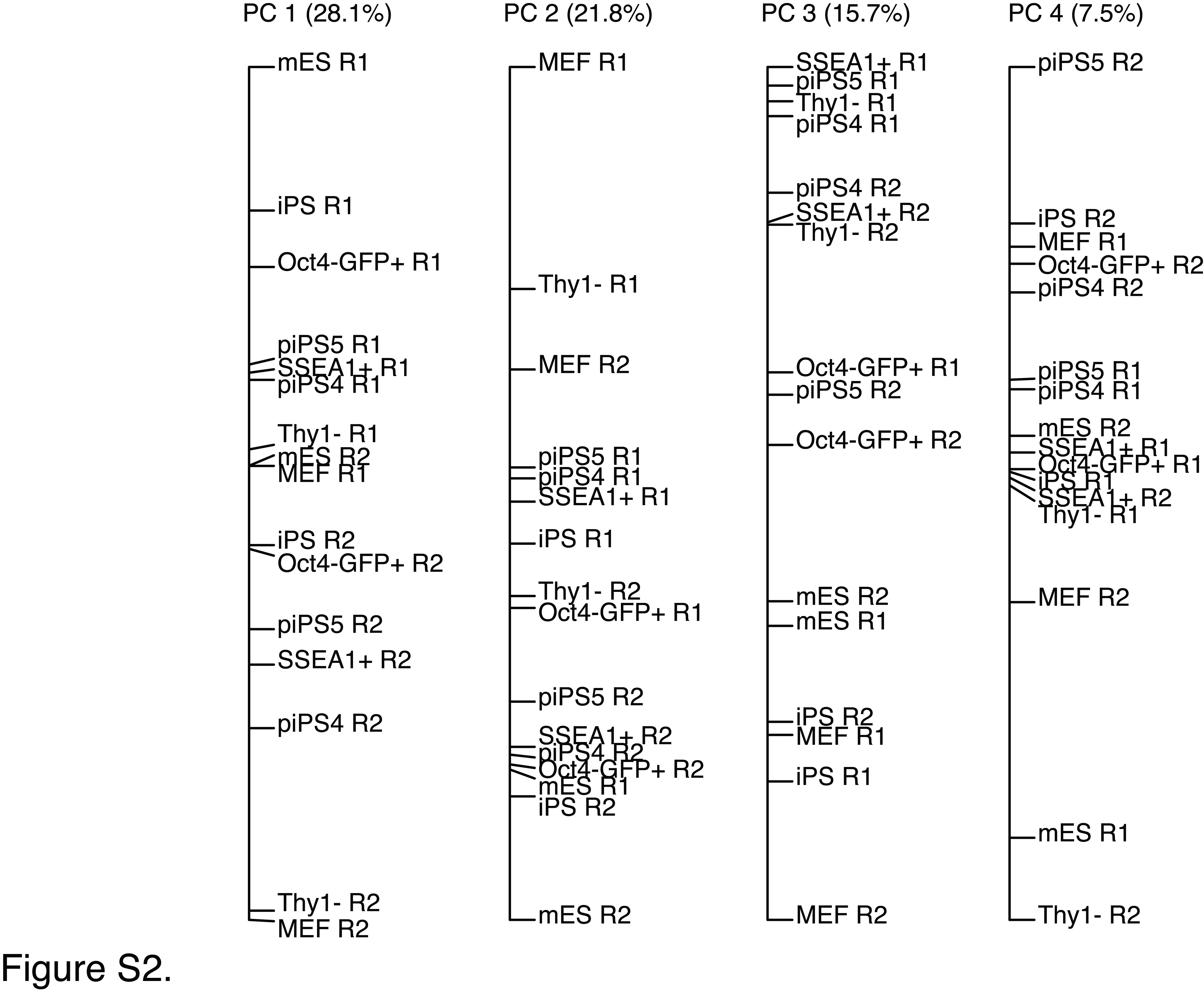


**Figure S2. First four principal components of PCA plotted one-dimensionally.**

The percent of variation represented by each is listed at the top of the figure, and the first four principal components altogether accounted for 73.1% of the variation in the dataset.


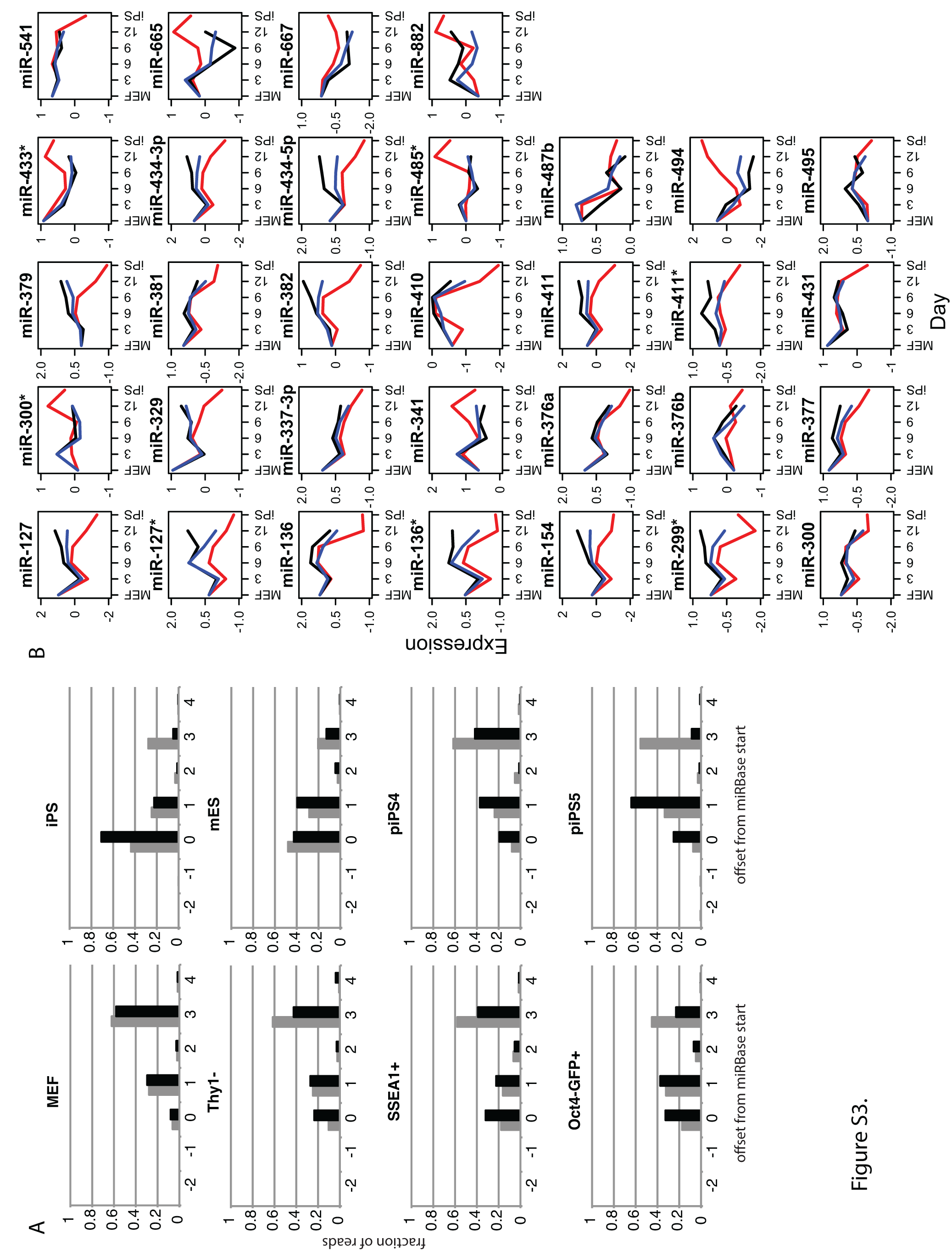


**Figure S3. Isomir variation and microarray re-analysis**

A) miR-485-3p isomir variation. Fraction of reads with varying 5’ start sites for replicates 1 (gray) and 2 (black); miRBase mature miRNA start site is 0. B) Log_2_ fold change of Thy1+ (black), Thy1– (blue) and successfully progressing reprogramming cells (red) from Polo et al.’s [5] microarray study. The first intermediate stage that Polo et al. sampled after inducing reprogramming was the SSEA1+ stage, so Thy1+ and Thy1– cells were subsets of cells that did not progress along the reprogramming trajectory, but maintained their Thy1– or Thy1+ and SSEA1- character throughout the experiment. Day 0 corresponds to MEFs at the initiation of reprogramming, days 3,6 and 9 are SSEA1+, day 12 is Oct4-GFP+ and the final sample is a characterized iPS line.


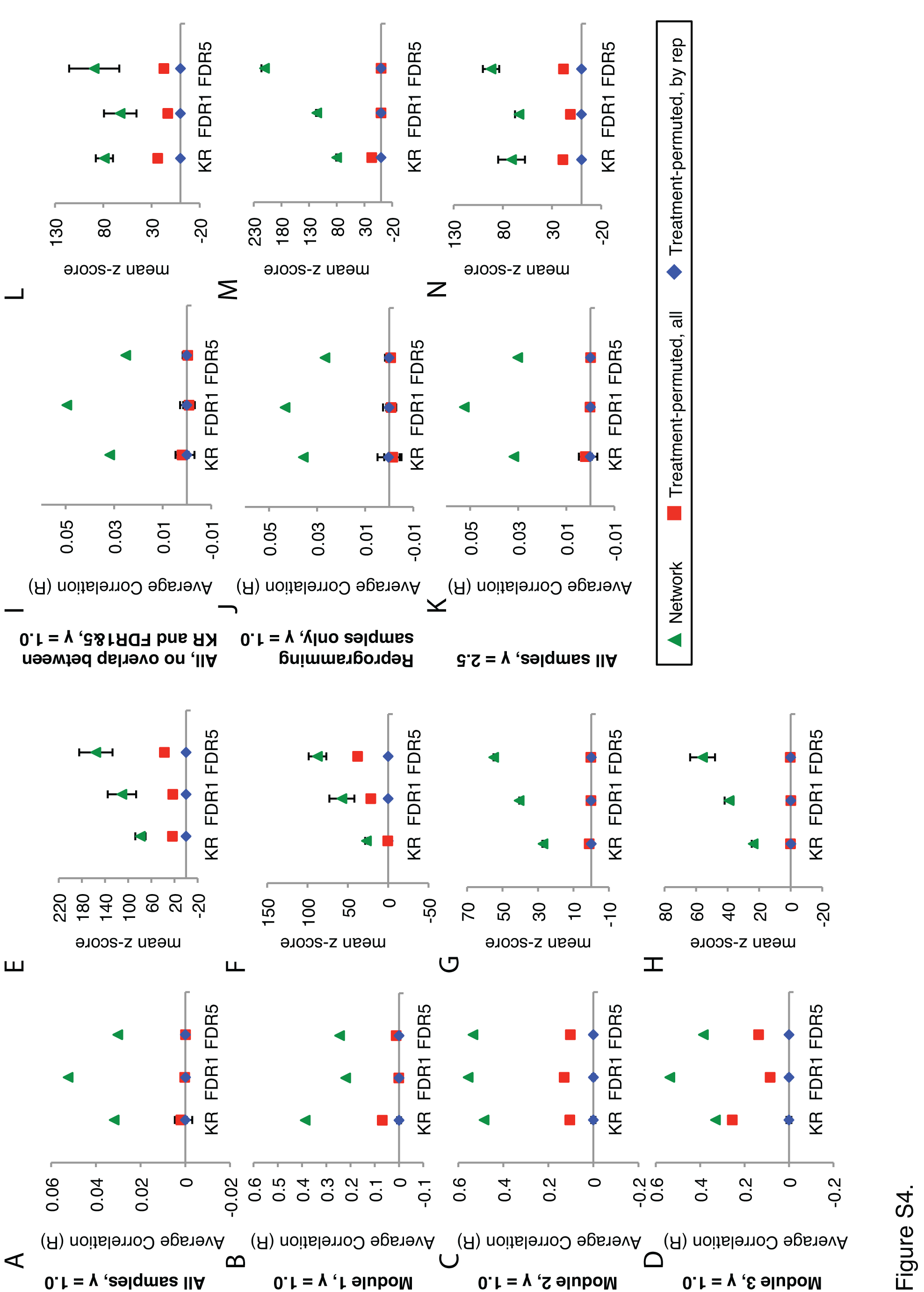


**Figure S4. Average correlation and partition similarity (mean z-score) for network analyses.**

Average correlation is calculated for 100 optimizations of each dataset and for two types of randomly permuted datasets:1000 datasets either randomly permuted across all treatments (treatment-permuted, all) or randomly permuted within replicates (treatment-permuted, by rep). For the within-module analyses, average correlation is expected to be higher in each dataset than in corresponding random-permuted datasets, and that was the case for all three modules. Mean z-scores are calculated between the representative partition of the actual data and the 1000 randomly permuted datasets of each type. For comparison, the z-score of the 100 optimizations of the network compared to the representative network of those 100 optimizations is shown (blue). Error bars are standard deviation. Average correlation and partition similarity are shown for. (A & E) all samples, γ=1.0. (B-H): subsets of each dataset (KR, FDR1, FDR5) corresponding to the three modules detected at γ=1; (I & L) FDR1 and FDR5 datasets modified to remove all miRNAs that overlap with the KR dataset (γ=1, KR dataset as in A & E shown for comparison), (J & M) KR and unmodified FDR1 and FDR5 datasets using reprogramming treatments only, and (C & F) KR and unmodified FDR1 and FDR5 datasets using γ=2.5.


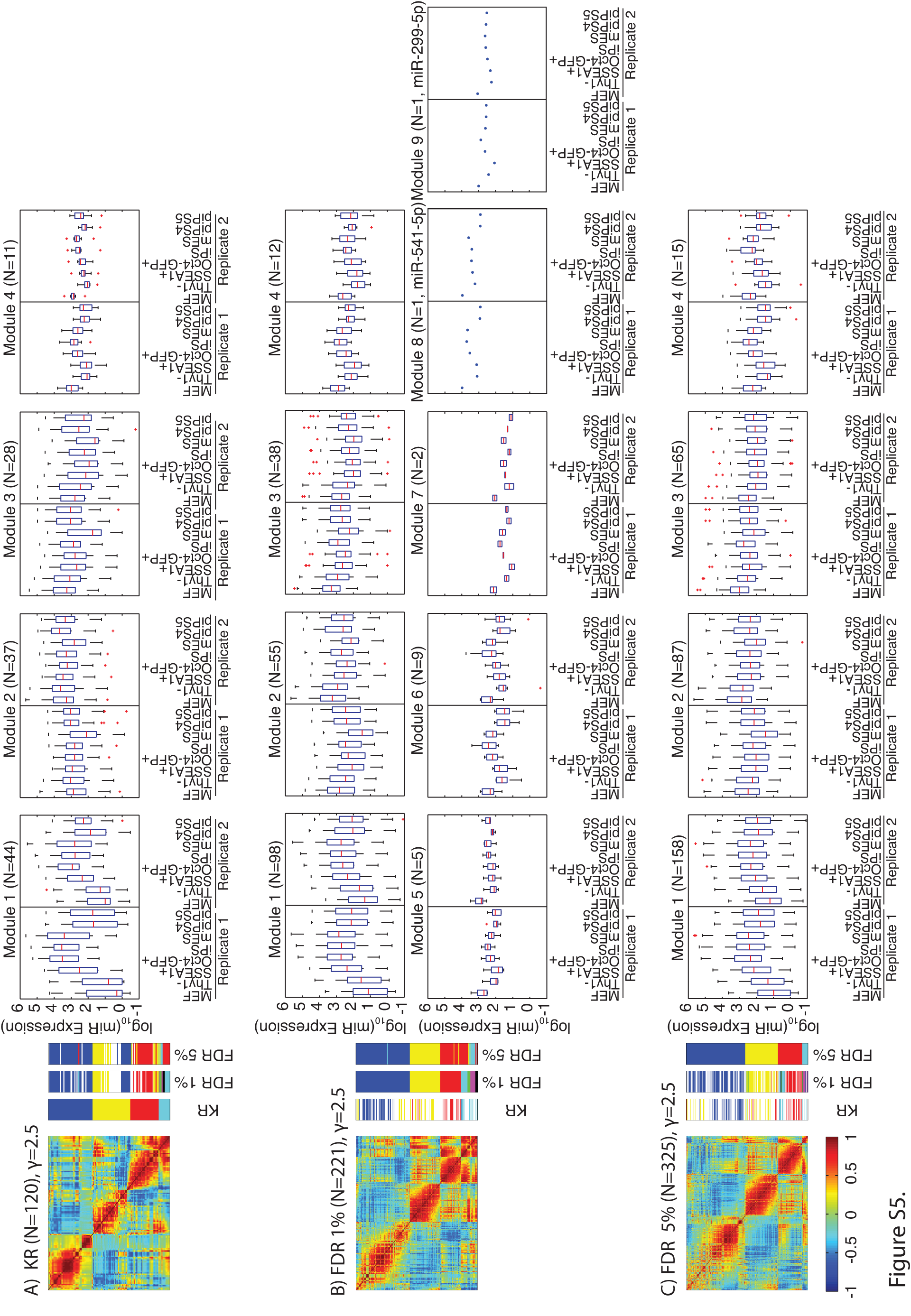


**Figure S5. Fine scale community structure uncovered at a larger value of the community resolution parameter (γ=2.5).**

For the A) KR, B) FDR1 and C) FDR5 datasets, a heatmap of the correlation matrix is shown on the left. Each row and column represent a single miRNA. Module assignments are from the representative partition. Colorbars indicate the module assignment for each miRNA in the heatmap to the left for each of the three datasets. Module 1 is blue, module 2 is yellow, module 3 is red, module 4 is cyan, module 5 is green, module 6 is magenta, and the single miRNA modules, modules 7 and 8, are black. Whisker plots show the log_10_(expression) of the miRNAs in the module.


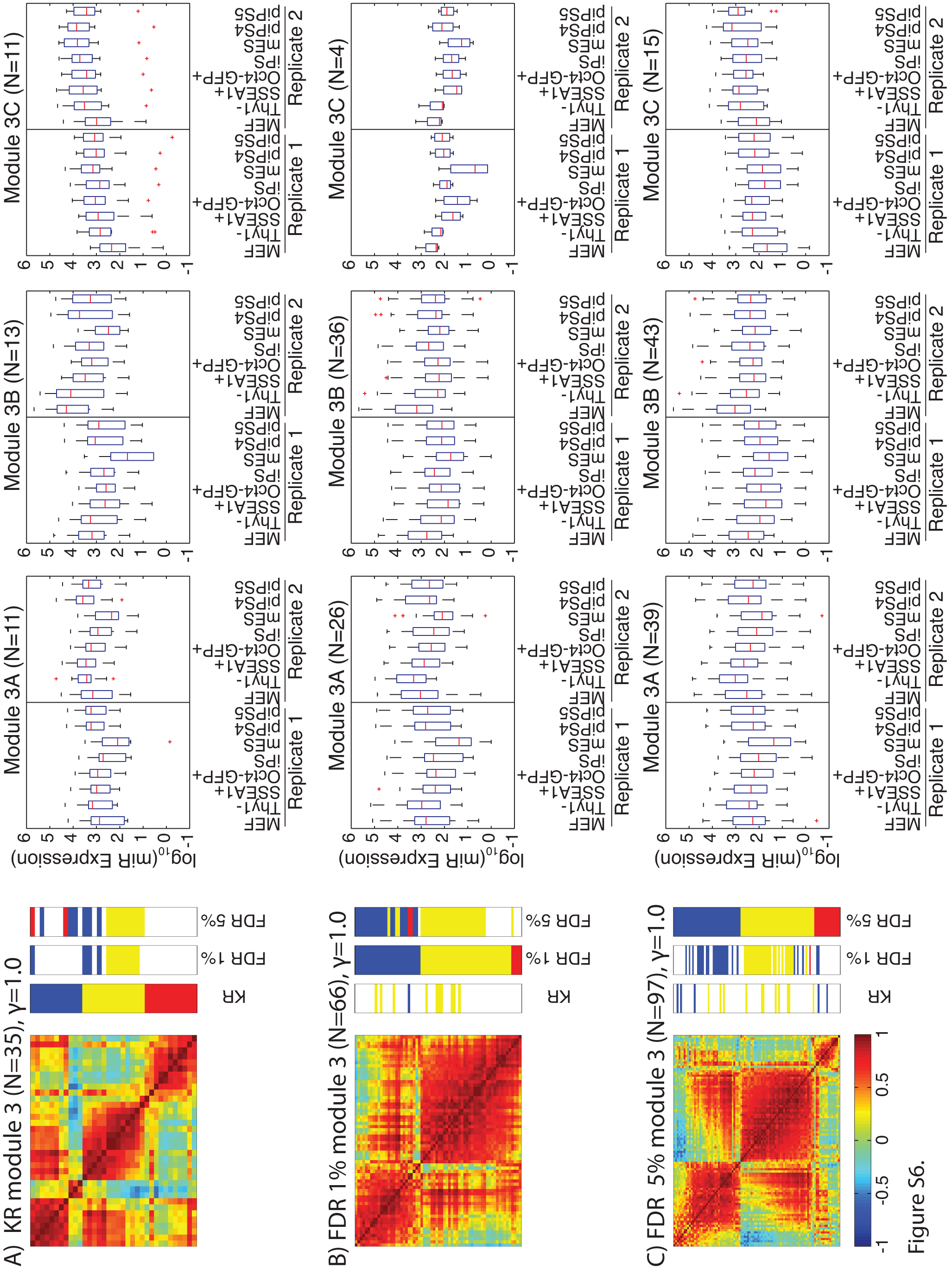


**Figure S6. Community structure of miRNAs found in module 3 of the representative partition of the original modular decomposition performed at gamma=1.**

For the A) KR, B) FDR1 and C) FDR5 datasets, the heatmap of the correlation matrix is shown on the left. Each row and column represent a single miRNA. Colorbars indicate the submodule assignment in each dataset of every miRNA in the heatmap to the left. Submodule 1 is blue, 2 is yellow, 3 is red, 4 is cyan and 5 is green. Not all miRNAs in each dataset are present in the other two datasets; miRNAs that are absent are white in the colorbar. Whisker plots show the log_10_(expression) of the miRNAs in the submodule.

**Supplementary Tables**

Table S1. EdgeR log_2_ fold changes between MEF, intermediate reprogramming stages, stem cells (labeled SC; Oct4-GFP+ newly reprogrammed cells, iPS, and mES lines combined), and piPS. Values are significant at an FDR of 5%, bold values are significant with an FDR of 1%. [Provided as an Excel file]

Table S2. EdgeR log_2_ fold changes in reprogramming. Values are significant at an FDR of 5%, bold values are significant with an FDR of 1%. log_2_ CPM is log_2_ of counts per million. [Provided as an Excel file]

Table S3. miRNAs that are known from the literature to be differentially expressed between MEFs and iPSCs or ESCs, and/or to enhance reprogramming. The five miRNAs in italics did not meet the basic abundance requirements for edgeR analysis and were not considered in further analyses, including the network analysis. [Provided as an Excel file]

**Table S4. Summary statistics for community structure analyses for all datasets.**

N=the number of miRNAs in each dataset; γ= the community resolution parameter; R=average correlation; Q=average modularity over all iterations or permutations; S=the number of modules detected for a given dataset.

|  |  | | |  |  |  |  |  | Randomly-permuted across all treatments^[[1]](#footnote-1)^ | | | | | Randomly-permuted across treatments within replicates^[[2]](#footnote-2)^ | | | | |
| --- | --- | --- | --- | --- | --- | --- | --- | --- | --- | --- | --- | --- | --- | --- | --- | --- | --- | --- |
| Dataset | N | | | γ | R | Q  (± SD) | S | Mean  z-score ^[[3]](#footnote-3)^  (± SD) | S (± SD) | R (± SD) | p^[[4]](#footnote-4)^ | Q  (± SD) | p4 | S (± SD) | R (± SD) | p4 | Q  (± SD) | p4 |
| KR | 120 | | | 1 | 0.032 | 0.364 ±0.006 | 3 | 75.4±10.8 | 5.60  ±0.74 | -1.79E-05  ±3.10E-03 | 0 | 0.235±0.0092 | 0 | 3.49±0.60 | 2.00E-03  ±2.70E-03 | 0 | 0.318  ±0.007 | 0 |
| FDR1 | 312 | | | 1 | 0.052 | 0.338 ±0.004 | 3 | 100.3±24.6 | 6.30  ±0.74 | -1.17E-04  ±1.60E-03 | 0 | 0.2121±0.0068 | 0 | 3.10±0.59 | 2.63E-04  ±1.40E-03 | 0 | 0.284  ±0.005 | 0 |
| FDR5 | 400 | | | 1 | 0.030 | 0.337 ±0.003 | 3 | 143.1±32.4 | 6.53  ±0.79 | -9.77E-06  ±1.10E-03 | 0 | 0.1986±0.0060 | 0 | 3.04±0.48 | -9.71E-05  ±9.47E-04 | 0 | 0.302  ±0.003 | 0 |
| miRNAs that overlap with KR dataset removed | | | | | | | |  |  |  |  |  |  |  |  |  |  |  |
| FDR1 | 136 | | | 1 | 0.050 | 0.325 ±0.005 | 3 | 56.5±18.4 | 5.80  ±0.73 | 1.88E-05  ±2.80E-03 | 0 | 0.2316±0.0085 | 0 | 3.18±0.58 | -8.85E-04  ±2.40E-03 | 0 | 0.295  ±0.007 | 0 |
| FDR5 | 223 | | | 1 | 0.025 | 0.327 ±0.004 | 4 | 87.7±34.7 | 6.21  ±0.72 | 4.10E-05  ±1.70E-03 | 0 | 0.2104±0.0068 | 0 | 2.79±0.59 | -3.73E-04  ±1.40E-03 | 0 | 0.309  ±0.005 | 0 |
| Reprogramming samples only | | | | | |  |  |  |  |  |  |  |  |  |  |  |  |  |
| KR | 120 | | 1 | | 0.036 | 0.401 ±0.005 | 3 | 77.5±9.4 | 4.87  ±0.62 | 2.73E-04  ±4.60E-03 | 0 | 0.2838±0.0108 | 0 | 4.06±0.53 | -1.50E-03  ±3.60E-03 | 0 | 0.327  ±0.008 | 0 |
| FDR1 | 221 | | 1 | | 0.043 | 0.402  ±3.18E-06 | 3 | 116.2±2.1 | 5.40  ±0.61 | 6.19E-05  ±2.50E-03 | 0 | 0.2726±0.0078 | 0 | 4.81±0.62 | -8.14E-04  ±2.10E-03 | 0 | 0.281  ±0.008 | 0 |
| FDR5 | 325 | | 1 | | 0.027 | 0.387  ±0.002 | 3 | 216.7±9.8 | 5.49  ±0.59 | 3.34E-05  ±1.70E-03 | 0 | 0.2615±0.0065 | 0 | 3.89±0.58 | -6.12E-04  ±1.40E-03 | 0 | 0.283  ±0.007 | 0 |
| Gamma=2.5 | | |  | |  |  |  |  |  |  |  |  |  |  |  |  |  |  |
| KR | 120 | | 2.5 | | 0.032 | 0.317  ±0.003 | 4 | 66.2±15.6 | 6.12  ±0.79 | 9.21E-05  ±3.00E-03 | 0 | 0.226±0.0092 | 0 | 5.12±0.70 | 2.10E-03  ±2.70E-03 | 0 | 0.314  ±0.007 | 2.28E-32 |
| FDR1 | 221 | | 2.5 | | 0.052 | 0.306  ±0.002 | 9 | 62.2±5.1 | 6.54  ±0.76 | -5.10E-05  ±1.60E-03 | 0 | 0.2085±0.0070 | 0 | 4.34±0.69 | 1.27E-04  ±1.40E-03 | 0 | 0.280  ±0.006 | 0 |
| FDR5 | 325 | | 2.5 | | 0.030 | 0.312  ±0.002 | 4 | 90.0±9.8 | 6.72  ±0.81 | -3.14E-05  ±1.10E-03 | 0 | 0.1963±0.0060 | 0 | 4.17±0.65 | -1.13E-04  ±9.36E-04 | 0 | 0.296  ±0.004 | 0 |
|  |  | |  | |  |  |  |  |  |  |  |  |  |  |  |  |  |  |
| Module 1 submodules | | | | |  |  |  |  |  |  |  |  |  |  |  |  |  |  |
| KR | 45 | | 1 | | 0.387 | 0.210  ±0.002 | 3 | 26.7±2.3 | 5.11  ±0.71 | 1.47E-05  ±8.20E-03 | 0 | 0.306±0.0137 | 0 | 4.78±0.68 | 6.90E-02  ±7.87E-03 | 0 | 0.155  ±0.198 | 0 |
| FDR1 | 97 | | 1 | | 0.220 | 0.268  ±0.008 | 4 | 51.5±16.8 | 5.26  ±0.67 | -4.09E-05  ±3.80E-03 | 0 | 0.2438±0.0100 | 0 | 3.67±0.57 | 9.73E-04  ±3.30E-03 | 0 | 0.294  ±0.009 | 0 |
| FDR5 | 145 | | 1 | | 0.245 | 0.241  ±0.001 | 4 | 85.9±10.6 | 5.59  ±0.73 | -1.50E-05  ±2.40E-03 | 0 | 0.2244±0.0083 | 1.38E-269 | 3.86±0.57 | 1.18E-02  ±2.40E-03 | 0 | 0.265  ±0.008 | 0 |
| Module 2 submodules | | | | |  |  |  |  |  |  |  |  |  |  |  |  |  |  |
| KR | 40 | | 1 | | 0.486 | 0.195  ±5.92E-05 | 2 | 27.1±0.5 | 4.74  ±0.70 | 2.11E-04  ±9.20E-03 | 0 | 0.305±0.140 | 0 | 4.46±0.63 | 1.05E-01  ±8.10E-03 | 0 | 0.260  ±0.014 | 0 |
| FDR1 | 58 | | 1 | | 0.556 | 0.135  ±1.81E-16 | 2 | 40.6  ±8.2E-13 | 5.15  ±0.72 | -1.99E-04  ±6.20E-03 | 0 | 0.284±0.0118 | 1.54  E-301 | 4.64±0.68 | 1.30E-01  ±6.40E-03 | 0 | 0.261  ±0.012 | 0 |
| FDR5 | 83 | | 1 | | 0.535 | 0.143  ±1.24E-04 | 3 | 55.0±0.2 | 5.33  ±0.72 | 2.06E-04  ±4.50E-03 | 0 | 0.256±0.0106 | 0 | 4.91±0.67 | 1.02E-01  ±4.00E-03 | 0 | 0.244  ±0.010 | 0 |
| Module 3 submodules | | | | |  |  |  |  |  |  |  |  |  |  |  |  |  |  |
| KR | | 35 | 1 | | 0.331 | 0.237  ±0.001 | 3 | 23.6±1.4 | 4.71  ±0.69 | 3.55E-04  ±1.10E-02 | 0 | 0.3177±0.0143 | 0 | 4.24±0.66 | 2.55E-01  ±9.10E-03 | 0 | 0.199  ±0.013 | 0 |
| FDR1 | | 66 | 1 | | 0.537 | 0.142  ±3.59E-04 | 3 | 38.3±3.6 | 5.59  ±0.75 | 4.57E-06  ±5.50E-03 | 0 | 0.2869±0.012 | 0 | 5.28±0.72 | 8.49E-02  ±5.60E-03 | 0 | 0.306  ±0.012 | 0 |
| FDR5 | | 97 | 1 | | 0.386 | 0.187  ±0.001 | 3 | 53.1±8.3 | 5.80  ±0.78 | 5.52E-05  ±3.80E-03 | 0 | 0.1874±0.0013 | 0 | 5.29±0.69 | 1.37E-01  ±3.80E-03 | 0 | 0.249  ±0.009 | 0 |

**Table S5. Comparisons between network partitions.**

For samples, ‘all’ means all 16 samples (2 replicates x 8 samples) were used, while ‘reprogramming only’ indicates only the 8 reprogramming samples (2 replicates x 4 samples) were used. See text for details. N = the number of miRNAs shared between the two datasets and used to calculate z-score.

| Samples | γ | comparison | N | z-score |
| --- | --- | --- | --- | --- |
| Comparisons between datasets | | |  |  |
| all | 1 | KR vs. FDR1 | 85 | 28.8 |
| all | 1 | KR vs. FDR5 | 102 | 48.8 |
| all | 1 | FDR1 vs. FDR5 | 221 | 99.8 |
| all | 2.5 | KR vs. FDR1 | 85 | 25.3 |
| all | 2.5 | KR vs. FDR5 | 102 | 29.9 |
| all | 2.5 | FDR1 vs. FDR5 | 221 | 74.3 |
| FDR1/FDR5, no overlap with KR, all | 1 | FDR1 vs. FDR5 | 136 | 67.4 |
| reprogramming only | 1 | KR vs. FDR1 | 85 | 43.7 |
| reprogramming only | 1 | KR vs. FDR5 | 102 | 48.4 |
| reprogramming only | 1 | FDR1 vs. FDR5 | 221 | 111.1 |
| submodule 1, all | 1 | KR vs. FDR1 | 33 | 21.4 |
| submodule 1, all | 1 | KR vs. FDR5 | 41 | 21.6 |
| submodule 1, all | 1 | FDR1 vs. FDR5 | 88 | 37.5 |
| submodule 2, all | 1 | KR vs. FDR1 | 20 | 8.5 |
| submodule 2, all | 1 | KR vs. FDR5 | 33 | 16.3 |
| submodule 2, all | 1 | FDR1 vs. FDR5 | 53 | 27.2 |
| submodule 3, all | 1 | KR vs. FDR1 | 11 | 1.3 |
| submodule 3, all | 1 | KR vs. FDR5 | 16 | 4.1 |
| submodule 3, all | 1 | FDR1 vs. FDR5 | 52 | 24.7 |
| Comparisons within datasets | | |  |  |
| FDR1, all | 1 | FDR1 vs. FDR1, no overlap with KR | 136 | 68.7 |
| FDR5, all | 1 | FDR5 vs. FDR5, no overlap with KR | 223 | 106.7 |
| KR, all | - | KR γ=1 vs. KR γ=2.5 | 120 | 72.3 |
| FDR1, all | - | FDR1 γ=1 vs. FDR1 vs. γ=2.5 | 221 | 87.1 |
| FDR5, all | - | FDR5 γ=1 vs. FDR5 vs. γ=2.5 | 325 | 96.6 |
| KR, all | 1 | KR all vs. KR reprogramming | 120 | 60.3 |
| FDR1, all | 1 | FDR1 γ=1 all vs. FDR1 γ=1 reprogramming | 221 | 77.9 |
| FDR5, all | 1 | FDR5 γ=1 all vs. FDR5 γ=1 reprogramming | 325 | 85.7 |

**Supplemental References**

1. Morin RD, O'Connor MD, Griffith M, Kuchenbauer F, Delaney A, Prabhu AL, Zhao Y, McDonald H, Zeng T, Hirst M, Eaves CJ, Marra MA: **Application of massively parallel sequencing to microRNA profiling and discovery in human embryonic stem cells**. *Genome Res* 2008, **18**:610–621.

2. Lengner CJ, Camargo FD, Hochedlinger K, Welstead GG, Zaidi S, Gokhale S, Schöler HR, Tomilin A, Jaenisch R: **Oct4 Expression Is Not Required for Mouse Somatic Stem Cell Self-Renewal**. *Cell Stem Cell* 2007, **1**:403–415.

3. Brambrink T, Foreman R, Welstead G, Lengner C, Wernig M, Suh H, Jaenisch R: **Sequential expression of pluripotency markers during direct reprogramming of mouse somatic cells**. *Cell Stem Cell* 2008, **2**:151–159.

4. Stadtfeld M, Maherali N, Breault D, Hochedlinger K: **Defining molecular cornerstones during fibroblast to iPS cell reprogramming in mouse**. *Cell Stem Cell* 2008, **2**:230–240.

5. Polo JM, Anderssen E, Walsh RM, Schwarz BA, Nefzger CM, Lim SM, Borkent M, Apostolou E, Alaei S, Cloutier J, Bar-Nur O, Cheloufi S, Stadtfeld M, Figueroa ME, Robinton D, Natesan S, Melnick A, Zhu J, Ramaswamy S, Hochedlinger K: **A molecular roadmap of reprogramming somatic cells into iPS cells.** *Cell* 2012, **151**:1617–1632.

6. Zhou H, Arcila ML, Li Z, Lee EJ, Henzler C, Liu J, Rana TM, Kosik KS: **Deep annotation of mouse iso-miR and iso-moR variation**. *Nucleic Acids Res* 2012, **40**:5864–5875.

7. Lewis BP, Burge CB, Bartel DP: **Conserved seed pairing, often flanked by adenosines, indicates that thousands of human genes are microRNA targets.** *Cell* 2005, **120**:15–20.

8. Conaco CC, Bassett DSD, Zhou HH, Arcila MLM, Degnan SMS, Degnan BMB, Kosik KSK: **Functionalization of a protosynaptic gene expression network.** *Proc Natl Acad Sci USA* 2012, **109 Suppl 1**:10612–10618.

9. Li Z, Yang C-S, Nakashima K, Rana TM: **Small RNA-mediated regulation of iPS cell generation**. *The EMBO Journal* 2011, **30**:823–834.

10. Samavarchi-Tehrani P, Golipour A, David L, Sung H-K, Beyer TA, Datti A, Woltjen K, Nagy A, Wrana JL: **Functional genomics reveals a BMP-driven mesenchymal-to-epithelial transition in the initiation of somatic cell reprogramming.** *Cell Stem Cell* 2010, **7**:64–77.

11. Houbaviy H, Murray M, Sharp P: **Embryonic stem cell-specific MicroRNAs**. *Developmental Cell* 2003, **5**:351–358.

12. Marson A, Levine SS, Cole MF, Frampton GM, Brambrink T, Johnstone S, Guenther MG, Johnston WK, Wernig M, Newman J, Calabrese JM, Dennis LM, Volkert TL, Gupta S, Love J, Hannett N, Sharp PA, Bartel DP, Jaenisch R, Young RA: **Connecting microRNA genes to the core transcriptional regulatory circuitry of embryonic stem cells.** *Cell* 2008, **134**:521–533.

13. Judson R, Babiarz J, Venere M, Blelloch R: **Embryonic stem cell–specific microRNAs promote induced pluripotency**. *Nat Biotechnol* 2009, **27**:459–461.

14. Li R, Liang J, Ni S, Zhou T, Qing X, Li H, He W, Chen J, Li F, Zhuang Q, Qin B, Xu J, Li W, Yang J, Gan Y, Qin D, Feng S, Song H, Yang D, Zhang B, Zeng L, Lai L, Esteban MA, Pei D: **A mesenchymal-to-epithelial transition initiates and is required for the nuclear reprogramming of mouse fibroblasts.** *Cell Stem Cell* 2010, **7**:51–63.

15. Melton C, Judson RL, Blelloch R: **Opposing microRNA families regulate self-renewal in mouse embryonic stem cells**. *Nature* 2010, **463**:621–626.

16. Anokye-Danso F, Trivedi CM, Juhr D, Gupta M, Cui Z, Tian Y, Zhang Y, Yang W, Gruber PJ, Epstein JA, Morrisey EE: **Highly efficient miRNA-mediated reprogramming of mouse and human somatic cells to pluripotency.** *Cell Stem Cell* 2011, **8**:376–388.

17. Choi YJ, Lin C-P, Ho JJ, He X, Okada N, Bu P, Zhong Y, Kim SY, Bennett MJ, Chen C, Ozturk A, Hicks GG, Hannon GJ, He L: **miR-34 miRNAs provide a barrier for somatic cell reprogramming.** *Nat Cell Biol*  2011, **13**:1353–1360.

18. Liao B, Bao X, Liu L, Feng S, Zovoilis A, Liu W, Xue Y, Cai J, Guo X, Qin B, Zhang R, Wu J, Lai L, Teng M, Niu L, Zhang B, Esteban MA, Pei D: **MicroRNA Cluster 302-367 Enhances Somatic Cell Reprogramming by Accelerating a Mesenchymal-to-Epithelial Transition**. *J Biol Chem* 2011, **286**:17359–17364.

19. Pfaff N, Fiedler J, Holzmann A, Schambach A, Moritz T, Cantz T, Thum T: **miRNA screening reveals a new miRNA family stimulating iPS cell generation via regulation of Meox2**. *Nature Publishing Group* 2011, **12**:1154–1160.

20. Yang CS, Li Z, Rana TM: **microRNAs modulate iPS cell generation**. *RNA* 2011, **17**:1451–1460.

21. Wang J, He Q, Han C, Gu H, Jin L, Li Q, Mei Y, Wu M: **p53-facilitated miR-199a-3p regulates somatic cell reprogramming.** *Stem Cells* 2012, **30**:1405–1413.

22. Yu X, Cohen DM, Chen CS: **miR-125b Is an Adhesion-Regulated microRNA that Protects Mesenchymal Stem Cells from Anoikis**. *Stem Cells* 2012, **30**:956–964.

23. Wilson KD, Venkatasubrahmanyam S, Jia F, Sun N, Butte AJ, Wu JC: **MicroRNA Profiling of Human-Induced Pluripotent Stem Cells**. *Stem Cells and Development* 2009, **18**:749–758.

24. Svoboda P, Flemr M: **The role of miRNAs and endogenous siRNAs in maternal-to-zygotic reprogramming and the establishment of pluripotency**. *Nature Publishing Group* 2010, **11**:590–597.

25. Houbaviy HB: **Characterization of a highly variable eutherian microRNA gene**. *RNA* 2005, **11**:1245–1257.

26. Porter MA, Onnela J-P, Mucha PJ: **Communities in networks**. *Notices of the AMS* 2009, **56**:1082–1097.

27. Fortunato S: **Community detection in graphs**. *Phys Rep* 2010, **486**:75–174.

28. Blondel VD, Guillaume JL, Lambiotte R, Lefebvre E: **Fast unfolding of communities in large networks**. *Journal of Statistical Mechanics: Theory and Experiment* 2008, **2008**:P10008.

29. Newman MEJ, Girvan M: **Finding and evaluating community structure in networks.** *Phys Rev E Stat Nonlin Soft Matter Phys* 2004, **69**:026113.

30. Newman MEJ: **Fast algorithm for detecting community structure in networks.** *Phys Rev E Stat Nonlin Soft Matter Phys* 2004, **69**:066133.

31. Traag VA, Bruggeman J: **Community detection in networks with positive and negative links.** *Phys Rev E* 2009, **80**:036115.

32. Gómez S, Jensen P, Arenas A: **Analysis of community structure in networks of correlated data**. *Phys Rev E* 2009, **80**:016114.

33. Fortunato S, Barthélemy M: **Resolution limit in community detection.** *Proc Natl Acad Sci USA* 2007, **104**:36–41.

34. Bassett DS, Porter MA, Wymbs NF, Grafton ST, Carlson JM, Mucha PJ: **Robust detection of dynamic community structure in networks**. *Chaos* 2013, **23**:013142.

35. Traud AL, Mucha PJ, Porter MA: **Social structure of Facebook networks**. *Physica A: Statistical Mechanics and its Applications* 2012, **391**:4165–4180.

36. Mendell JT: **miRiad roles for the miR-17-92 cluster in development and disease.** *Cell* 2008, **133**:217–222.

1. 1000 randomly-permuted datasets created by permuting expression data for each miRNA across all treatments [↑](#footnote-ref-1)
2. 1000 randomly-permuted datasets created by permuting expression data for each miRNA across treatments within each replicate [↑](#footnote-ref-2)
3. Mean z-score between 100 optimizations of the algorithm [↑](#footnote-ref-3)
4. two-tailed t-test [↑](#footnote-ref-4)
